# Insects and associated arthropods analyzed during medicolegal death investigations in Harris County, Texas, USA: January 2013- April 2016

**DOI:** 10.1371/journal.pone.0179404

**Published:** 2017-06-12

**Authors:** Michelle R. Sanford

**Affiliations:** Harris County Institute of Forensic Sciences, Houston, TX, United States of America; North Carolina State University, UNITED STATES

## Abstract

The application of insect and arthropod information to medicolegal death investigations is one of the more exacting applications of entomology. Historically limited to homicide investigations, the integration of full time forensic entomology services to the medical examiner’s office in Harris County has opened up the opportunity to apply entomology to a wide variety of manner of death classifications and types of scenes to make observations on a number of different geographical and species-level trends in Harris County, Texas, USA. In this study, a retrospective analysis was made of 203 forensic entomology cases analyzed during the course of medicolegal death investigations performed by the Harris County Institute of Forensic Sciences in Houston, TX, USA from January 2013 through April 2016. These cases included all manner of death classifications, stages of decomposition and a variety of different scene types that were classified into decedents transported from the hospital (typically associated with myiasis or sting allergy; 3.0%), outdoor scenes (32.0%) or indoor scenes (65.0%). Ambient scene air temperature at the time scene investigation was the only significantly different factor observed between indoor and outdoor scenes with average indoor scene temperature being slightly cooler (25.2°C) than that observed outdoors (28.0°C). Relative humidity was not found to be significantly different between scene types. Most of the indoor scenes were classified as natural (43.3%) whereas most of the outdoor scenes were classified as homicides (12.3%). All other manner of death classifications came from both indoor and outdoor scenes. Several species were found to be significantly associated with indoor scenes as indicated by a binomial test, including *Blaesoxipha plinthopyga* (Wiedemann) (Diptera: Sarcophagidae), all Sarcophagidae (including *B*. *plinthopyga*), *Megaselia scalaris* Loew (Diptera: Phoridae), *Synthesiomyia nudiseta* Wulp (Diptera: Muscidae) and *Lucilia cuprina* (Wiedemann) (Diptera: Calliphoridae). The only species that was a significant indicator of an outdoor scene was *Lucilia eximia* (Wiedemann) (Diptera: Calliphoridae). All other insect species that were collected in five or more cases were collected from both indoor and outdoor scenes. A species list with month of collection and basic scene characteristics with the length of the estimated time of colonization is also presented. The data presented here provide valuable casework related species data for Harris County, TX and nearby areas on the Gulf Coast that can be used to compare to other climate regions with other species assemblages and to assist in identifying new species introductions to the area. This study also highlights the importance of potential sources of uncertainty in preparation and interpretation of forensic entomology reports from different scene types.

## Introduction

While forensic entomology has a broad scope, one of its most challenging applications is to medicolegal death investigations. The application of the information provided by insects and arthropods associated with death investigations can take many forms ranging from direct association with cause of death (e.g. insect sting allergy), to information related to decedent travel history (e.g. ticks traveling with their host decedents), to the of time of insect colonization (TOC) estimate that can be used to approximate the post-mortem interval (PMI; [1]). The use of insect colonization and development in estimating the PMI is one of the most well-known applications of forensic entomology in the medicolegal setting [2]. When one starts to investigate the insects associated with a broad range of decedents, scenes and manners of death one starts to appreciate the breadth of forensic entomology casework opportunities. One of the first steps to understanding these cases is the identification of the insects involved in colonizing human remains in a given geographic location, their seasonality and the characteristics of the scenes where they are found.

Forensic entomology surveys of different geographic locations are often performed as a first step in establishing baseline data (e.g. [3–5]). However, these types of studies are often based on trapping adult flies or using animal models, and are not typically based on casework information. In Texas, survey information specific to forensically important insects is rare with only a few published surveys available from Central Texas [5,6]. Blow fly (Diptera: Calliphoridae) trapping studies were prevalent prior to and immediately following the screwworm eradication program but the focus of these efforts was mainly to detect *Cochliomyia hominivorax* (Coquerel) (Diptera: Calliphoridae) [7–9]. A series of experiments examining the interaction between the newly introduced *Chrysomya* sp. (Diptera: Calliphoridae) into the United States also recorded the presence of several blow flies in Central parts of Texas with traps as well [10,11] but the focus of these studies was not primarily for survey purposes.

Species records and trends related to casework have been published in other geographic areas. In North America these types of surveys have included the Hawaiian island of Oahu [12,13] and Western Canada [14]. These types of data are rarely published but are exceedingly important to understanding the local fauna important to casework in different geographic locations especially when practitioners may be reliant upon old and restricted taxonomic keys, which may not include all the species local to the area. To date no published data relating casework and the local forensically important insects in Texas or specifically in Harris County has been recorded. In this study the species, trends and seasonality of the forensically important insects associated with casework are reported for Harris County, Texas, USA. These data will aid in not only guiding identification and tracking of new species in the area, but also in guiding research questions into the species of importance in this geographic location. This study also allows for comparison with previous studies, particularly with respect to larger scale trends in insect colonization patterns such as indoor and outdoor scenes, seasonality and introduced species.

## Materials and methods

### Casework

**Fig 1:**
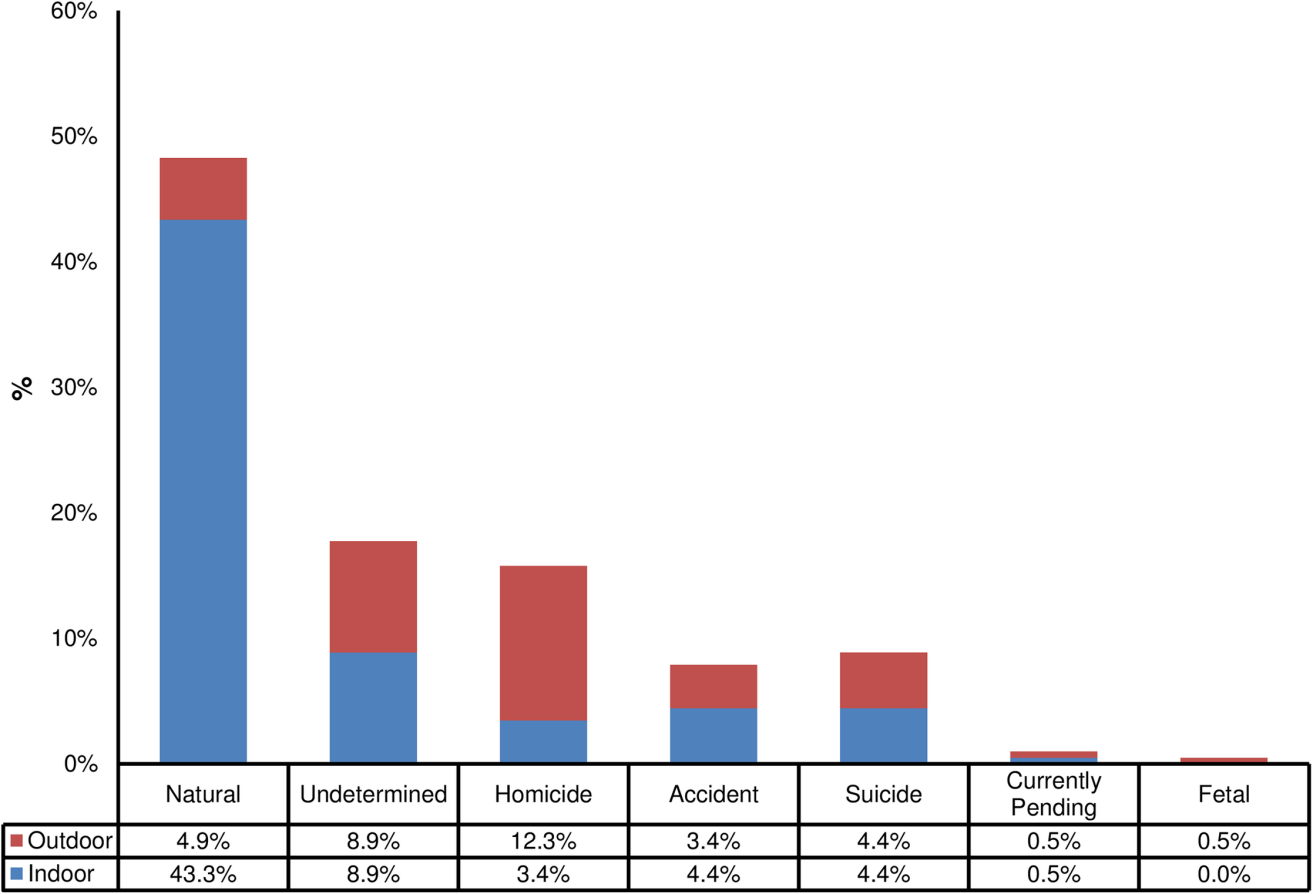
Forensic entomology cases by official manner of death and scene location. Percentage of forensic entomology cases over the study period (January 2013—April 2016) by official manner of death classification, showing the proportion of cases where the scene was located indoors (blue) or outdoors (red). (N = 203).

Cases involving insect or related arthropod specimens collected and analyzed during the course of medicolegal death investigations performed by the Harris County Institute of Forensic Sciences (HCIFS) as dictated under Texas Code of Criminal Procedure 49.25 [15], for the period of January 2013 through April 2016 (N = 203 of the 206 forensic entomology cases handled for this period) were included in this dataset. Three cases were not included in the analysis as they consisted of either review of an old case report (n = 1) or were reports on the absence of insect colonization (n = 2). All cases described here involved the collection of specimens at the scene or during the autopsy and occasionally at both locations (Fig 1). The procedure for specimen collection follows standard operating procedures developed for the office which are based on several texts and publications regarding insect collection, preservation and analysis [1,16–19]. Briefly, this includes collection of representative insect and/or related arthropod specimens from representative locations on the body and scene representing the assumed oldest observed life stages associated with the body. In order to increase the likelihood that the primary colonizing insects are collected, the body and surroundings at the scene are searched for the next most advanced life stage. For example if larvae are found pupae will be searched for or if pupae are found pupal exuvia will be searched for to attempt to collect the most developmentally advanced life stages available upon which to base an estimate of insect age. The number of specimens varies depending upon the number of insects present, the type of specimens (e.g. adult beetles typically collected individually vs. larval flies collected in samples of 10–20 per aggregation) and life stages available (e.g. pupae or newly emergent adults may be rare). Collected specimens are divided into representative portions, preserved by the most appropriate method (e.g. hot water kill followed by 70% ethanol for larval fly specimens [20]) and reared to the adult stage, if possible, for confirmation of identification. The identification of the immatures and resulting adults are then combined to provide as clear a picture as possible of the colonizing insect species in order to apply the correct insect species development dataset(s).

During the course of each scene investigation, a temperature and humidity recording was made using a combined thermometer-hygrometer (Fisher Scientific Education™, S66279, Fisher Scientific, Pittsburgh, PA, USA). Scene conditions such as location of the body with respect to shade or sun outdoors or thermostat settings indoors were also recorded to assist in the assessment of temperature modifications at the scene that might influence differences in temperature from the nearest weather station. Data were collected for ADH calculations from the nearest National Oceanic and Atmospheric Administration (NOAA) quality controlled weather station for each scene. Harris county has five quality controlled weather stations (at airport codes: IAH, HOU, MCJ, DWH and EFD) and four located adjacent to the county (at airport codes: SGR, LVJ, AXH and TME). To estimate a rough distance between the scene and nearest weather station, each scene location was plotted on a Google™ Earth Pro map containing markers for each weather station and measured using the measurement tool [21]. This rough measurement was used to determine the nearest weather station. The average distance to the nearest weather station based on these rough approximations was 12.7 km (+/-6.3 km standard deviation; minimum = 0.4 km; maximum = 18.4 km; N = 142 scenes).

Identification of specimens was based on morphology using life stage and taxon appropriate taxonomic keys and literature, which are referenced in each individual report and the most frequently used publications are presented in S1 Table. Specialized keys and consultation with taxonomic experts are applied as needed depending upon the specific case. Dr. T. Whitworth (Washington State University) also generously provided the HCIFS with a small reference collection of adult blow flies (Diptera: Calliphoridae) in 2013 to facilitate identification of adult Calliphoridae specimens.

Cases that require only identification of specimens, such as for stinging insect cases, may require only identification and reporting, but a majority of cases require further analysis to estimate the age of the collected specimens and to provide a TOC estimate. Larval Diptera and Coleoptera specimens were measured for length and each collected species is photographed for representative identifying features using a Keyence VHX-600 digital microscope with an attached VHX-S15E used as needed to stack images to enhance depth of field (Keyence Corp., Itasca, IL, USA). The estimated age of the larvae was calculated using the accumulated degree hours (ADH) method [1,22] and taxon appropriate published development data for each species using either published length data and/or development stage. Many scenes were located on private property with access granted by law enforcement as part of the death investigation as per Texas Code of Criminal Procedures 49.25. No protected species were collected. The complete TOC estimate and report for the specimens are included with the full autopsy report for each case involving the collection of entomology specimens.

### Statistics

Basic statistics were calculated for the forensic entomology cases where insects were collected from 2013 through April of 2016 for the 203 cases. Basic statistics (e.g. mean, standard deviation, count) were calculated for scene location (indoor, outdoor or hospital), month of scene investigation, length of TOC estimate, ambient scene temperature (°C), ambient scene relative humidity (%) (Table 1) and the presence of insect species collected for each case (Table 2). The data for the continuous variables (ambient scene temperature, relative humidity and the absolute value of the estimated TOC length) were subjected to normality check using the Shapiro-Wilk test and transformation was attempted by using ln(x+1) transformation (S1 File). Additionally the boxplot function in R Commander was used to evaluate the potential for outliers [23]. The tests indicated a failure to satisfy normality and several outliers, therefore a Kruskal-Wallis Χ^2^ test was implemented (S2 File) on the transformed data in order to satisfy the assumption that the data were represented by the same distribution [24]. An exact binomial test for categorical variable, scene location, for species, which were collected in five or more cases, was used to calculate the probability of encountering a species indoors, assuming a binomial distribution for the data. All statistical calculations and tests were computed using Microsoft Excel® and R 3.3.3 [25] using the R Commander package [23] and RStudio [26]. Box plots illustrating the median and associated variation for each continuous variable by category and violin plots illustrating the data for species evaluated with the binomial test were created using the R packages ggplot2 [27], RColorBrewer [28]and extrafont [29]. The de-identified raw data have been provided in S3 File and additional figures for the non-significant results have been provided in the supplemental information as well (S1–S4 Figs).

**Table 1:** Average environmental factors by collected species.

Species/group	N In	N Out	Avg. Ambient Temperature (+/-st.dev.)	Avg. Ambient Relative Humidity (+/-st.dev.)	Avg. TOC (days) (+/-st.dev.)
**Diptera: Sarcophagidae**	**85**	**8**	25.8°C (5.1)	50.8% (14.8)	9.7d (12.1)
- *Blaesoxipha plinthopyga*^*1*^	**37**	**0**^**1**^	25.7°C (4.4)	49.6% (13.7)	9.8d (9.5)
**Diptera: Phoridae**	**60**	**2**	24.1°C (4.5)	53.0% (16.5)	8.8d (7.6)
- *Megaselia scalaris*	**59**	**2**	24.1°C (4.5)	53.0% (16.5)	8.8d (7.6)
- *Dorniphora cornuta*^2^	**1**	**0**	16.6°C (unk)	56% (unk)	12d (unk)
**Diptera: Calliphoridae**	**64**	**56**	26.9°C (7.2)	53.5% (19.1)	11.3d (18.2)
- *Phormia regina*	**12**	**8**	22.4°C (7.2)	47.4% (18.0)	8.3d (10.8)
- *Cochliomyia macellaria*	**17**	**20**	29.2°C (6.7)	54.3% (16.5)	4.9d (6.5)
- *Chrysomya rufifacies*	**34**	**36**	27.7°C (7.8)	52.4% (21.7)	14.5d (21.2)
- *Chrysomya megacephala*	**21**	**19**	28.0°C (6.6)	55.6% (19.6)	3.6d (3.4)
- *Lucilia eximia*	**3**	**17**	29.2°C (6.6)	56.3% (21.8)	2.2d (2.8)
- *Lucilia cuprina*	**8**	**1**	27.4°C (7.5)	45.1% (10.9)	4.7d (1.9)
- *Lucilia sericata*	**1**	**0**	unk	unk	2.0 (unk)
- *Calliphora vomitoria*	**0**	**2**	21.2°C (10.3)	44.5% (4.9)	9.5d (3.5)
- *Calliphora livida*	**0**	**1**	15.2°C (8.8)	47% (12.7)	0 (unk)
- *Calliphora coloradensis*	**0**	**1**	6.1°C (unk)	75% (unk)	30d (unk)
- *Calliphora vicina*	**1**	**0**	25°C (unk)	36% (unk)	8d (unk)
**Diptera: Muscidae**	**20**	**14**	24.9°C (8.7)	52.2% (22.8)	19.6d (21.4)
- *Synthesiomyia nudiseta*	**9**	**1**	22.7°C (6.8)	50.6% (21.4)	17.4d (22.9)
- *Musca domestica*	**4**	**1**	26.4°C (8.2)	33.8% (20.3)	8.8d (8.2)
- *Hydrotea sp*.	**7**	**10**	26.7°C (11.2)	57.7% (25.4)	21.1d (22.8)
- *Fannia scalaris*	**0**	**1**	30.6°C (unk)	64% (unk)	11d (unk)
**Diptera: Piophilidae**					
- *Piophila casei*	**2**	**4**	26.2°C (9.3)	53.0% (21.1)	21.1d (37.6)
**Diptera: Stratiomyidae**					
* - Hermetia illucens*	**2**	**6**	23.3°C (10.0)	57.6% (20.6)	53.0d (33.3)
**Coleoptera: Dermestidae**	**8**	**11**	25.5°C (5.5)	57.5% (16.4)	29.9d (25.6)
**Coleoptera: Nitidulidae**					
- *Omosita* sp.	**0**	**5**	25.6°C (4.7)	67.0% (30.4)	36.8d (21.5)
**Coleoptera: Histeridae**^**3**^	**1**	**3**	32.0°C (6.5)	54.4% (25.6)	5.2d (4.3)
**Coleoptera: Silphidae**^**3**^	**0**	**2**	16.8°C (4.1)	63.0% (21.2)	5.0d (2.8)
**Coleoptera: Cleridae**^**3**^	**3**	**0**	27.5°C (unk)	83% (unk)	10d (unk)
**Hymenoptera: Apidae**					
- *Apis mellifera*^**3**^	**0**	**1**	29.8°C (unk)	39% (unk)	n/a
**Hymenoptera: Formicidae**					
- *Solenopsis invicta*^**3**^	**1**	**2**	34.7°C (2.3)	42.7% (13.3)	2d (unk)
**Hymenoptera: Chalcididae**					
- *Brachymeria fonscolombei*	**1**	**0**	31.1°C (unk)	60% (unk)	16d (unk)
**Hymenoptera: Pteromalidae**					
- *Nasonia vitripennis*	**0**	**1**	34.9°C (unk)	32% (unk)	43d (unk)
**Acari: Astigmatidae**	**0**	**3**			
- *Myianoetus muscuarum*^*4*^	**0**	**2**	19.2°C (2.0)	51.3% (30.5)	22.3d (2.1)
**Heteroptera: Cimicidae**					
- *Cimex lectularis*^**3**^	**1**	**0**	33.5°C (unk)	54% (unk)	18d (unk)
**Isopoda**^**3**^	**0**	**3**	28.5°C (17.6)	47.5%(37.5)	40.3d (51.1)

Number of cases coming from indoor and outdoor scene investigations with the average observed ambient scene temperature (°C) relative humidity (%) and the average maximum estimated time of colonization (TOC) for selected species collected in Harris County from January 2013 through April 2016 (N = 203).

^1^Confirmation of this species is based on the appearance of male genitalia from reared adult males and this could be an underestimate of actual abundance for this species because reared males may not always be obtained.

^2^Identification of this specimen is based on a single larva and is tentatively identified to the species level.

^3^These species may be regularly observed at scenes but not regularly collected and reported upon because they are not typically related to time of colonization estimation.

^4^This species was recently associated with *Synthesiomyia nudiseta*.

**Table 2:** Species list by scene type, month and year.

	**N**		**Jan**	**Feb**	**Mar**	**Apr**	**May**	**Jun**	**Jul**	**Aug**	**Sep**	**Oct**	**Nov**	**Dec**
**Scene Type: Hospital**	**6**													
**Species**														
**Diptera: Calliphoridae**	**3**													
- *Chrysomya rufifacies*	**1**							A						
- *Lucilia eximia*	**2**							A	B					
**Hymenoptera: Apidae**														
- *Apis mellifera*	**2**					A						B		
**Pthiraptera: Pulicidae**														
- *Pediculus humanus*	**1**							A						
**Acari: Ixodidae**														
- *Amblyomma imitator* or *cajennense* (nymph)	**1**									A				
**Scene Type: Scene**	**N** **In**	**N** **Out**	**Jan**	**Feb**	**Mar**	**Apr**	**May**	**Jun**	**Jul**	**Aug**	**Sep**	**Oct**	**Nov**	**Dec**
**Diptera: Sarcophagidae**	**85**	**8**	ABCD	ABCD	ABCD	ABCD	ABC	ABC	BC	ABC	ABC	ABC	ABC	ABC
- *Blaesoxipha plinthopyga*^*1*^	**37**	**0**^**1**^	BC	BC	BCD	C	AB	C	C	BC	AC	AC	AB	A
**Diptera: Phoridae**	**60**	**2**												
- *Megaselia scalaris*	**59**	**2**	ABC	ABCD	ABC	ACD	AB	ABC	BC	AC	ABC	ABC	ABC	ABC
- *Dorniphora cornuta*^2^	**1**	**0**												B
**Diptera: Calliphoridae**	**64**	**56**	AD	ACD	ABCD	ABCD	ABC	ABC	ABC	ABC	ABC	ABC	ABC	ABC
- *Phormia regina*	**12**	**8**	D	ACD	ABCD	ABCD		B	C			C		ABC
- *Cochliomyia macellaria*	**17**	**20**	D	A	D	ABCD	BC	ABC	ABC	C	BC	B		C
- *Chrysomya rufifacies*	**34**	**36**	AD	AC	ACD	D	AC	ABC	ABC	ABC	ABC	AC	BC	ABC
- *Chrysomya megacephala*	**21**	**19**		A	AD	ACD		ABC	ABC	AC	ABC	ABC	A	ABC
- *Lucilia eximia*	**3**	**17**			D	B	AC	AB		C	AB		ABC	B
- *Lucilia cuprina*	**8**	**1**			C	ABD	A					B		A
- *Lucilia sericata*	**1**	**0**				A								
- *Calliphora vomitoria*	**0**	**2**		A										B
- *Calliphora livida*	**0**	**1**	D	C										
- *Calliphora coloradensis*	**0**	**1**		C										
- *Calliphora vicina*	**1**	**0**			D									
**Diptera: Muscidae**	**20**	**14**	B	C	AB	ABC	AB	A	BC	B	C	C	BC	AB
- *Synthesiomyia nudiseta*	**9**	**1**	BD	CD	D		AB		C	B		C	BC	
- *Musca domestica*	**4**	**1**			A	AB	A							A
- *Hydrotea sp*.	**7**	**10**	D	CD	BD	ABC		A	BC	B	C	C	C	B
- *Fannia scalaris*	**0**	**1**						A						
**Diptera: Piophilidae**														
- *Piophila casei*	**2**	**4**				BD	A	A	C				C	
**Diptera: Stratiomyidae**														
- *Hermetia illucens*	**2**	**6**		A					BC			C		BC
**Diptera: Anthomyiidae**	**0**	**1**	A											
**Diptera: Sepsidae**	**0**	**1**	A											
**Diptera: Drosophilidae**	**1**	**0**												C
**Diptera: Chironomidae**	**0**	**1**												
- *Goelichironomus holoprasinus*	**0**	**1**									B			
- *Polypedlium flavum*	**0**	**1**									B			
- *Chironomus* sp.	**0**	**1**									B			
**Diptera: Tachnidae**	**0**	**1**									C			
**Diptera: Psychodidae**														
- *Psychoda* sp.	**0**	**1**							C					
**Coleoptera: Dermestidae**	**8**	**11**	AD	A	D	BD	AB	A	ABC	BC		C	BC	A
- *Dermestes maculatus*	**6**	**7**				D	AB	A	ABC	B	C	C	C	A
**Coleoptera: Nitidulidae**														
- *Omosita* sp.	**0**	**5**						A	C			C	C	A
**Coleoptera: Histeridae**^*3*^	**1**	**3**			A		A	A		A				
**Coleoptera: Silphidae**^*3*^	**0**	**2**		A										B
**Coleoptera: Cleridae**^*3*^	**3**	**0**						A						
**Coleoptera: Staphylinidae**	**0**	**1**		D										
**Coleoptera: Tenebrionidae**	**0**	**1**					A							
**Coleoptera: Carabidae**	**0**	**1**								B				
**Coleoptera: Elateridae**	**0**	**2**							C					B
**Hymenoptera: Apidae**														
- *Apis mellifera*	**0**	**1**		B										
**Hymenoptera: Formicidae**														
- *Solenopsis invicta*	**1**	**2**							AB	C				
- *Nylanderia fulva*	**0**	**1**				D								
**Hymenoptera: Chalcididae**														
- *Brachymeria fonscolombei*	**1**	**0**							B					
**Hymenoptera: Pteromalidae**														
- *Nasonia vitripennis*	**0**	**1**		D										
**Dictyoptera: Blatellidae**														
- *Blatella germanica*^*3*^	**1**	**0**											B	
**Lepidoptera**	**1**	**2**							C		C		B	
**Orthoptera: Gryllidae**	**0**	**1**								C				
**Orthoptera: Cicadellidae**	**0**	**1**												B
**Nematoda**	**1**	**1**		C		D			B					
**Acari: Astigmatidae**	**0**	**3**		C										C
- *Myianoetus muscuarum*	**0**	**2**		C										
**Acari: Macrochelidae**	**0**	**1**												A
**Heteroptera: Cimicidae**														
- *Cimex lectularis*^*3*^	**1**	**0**								C				
**Annelida**^**3**^	**0**	**1**												C
**Diplopoda**^**3**^	**0**	**1**		C										
**Isopoda**^**3**^	**0**	**3**				C			C					B

A list of insect and arthropod species collected during selected medicolegal death investigations handled by the Harris County Institute of Forensic Sciences from January 2013 through April 2016. The month of recorded collection is indicated by the letters A for 2013, B for 2014, C for 2015 and D for 2016.

^1^Confirmation of this species is based on the appearance of male genitalia from reared adult males and this could be an underestimate of actual abundance for this species because reared males may not always be obtained.

^2^Identification of this specimen is based on a single larva and is tentatively identified to the species level.

^3^These species may be regularly observed at scenes but not regularly collected and reported upon because they are not typically related to time of colonization estimation.

## Results and discussion

### Casework

**Fig 2:**
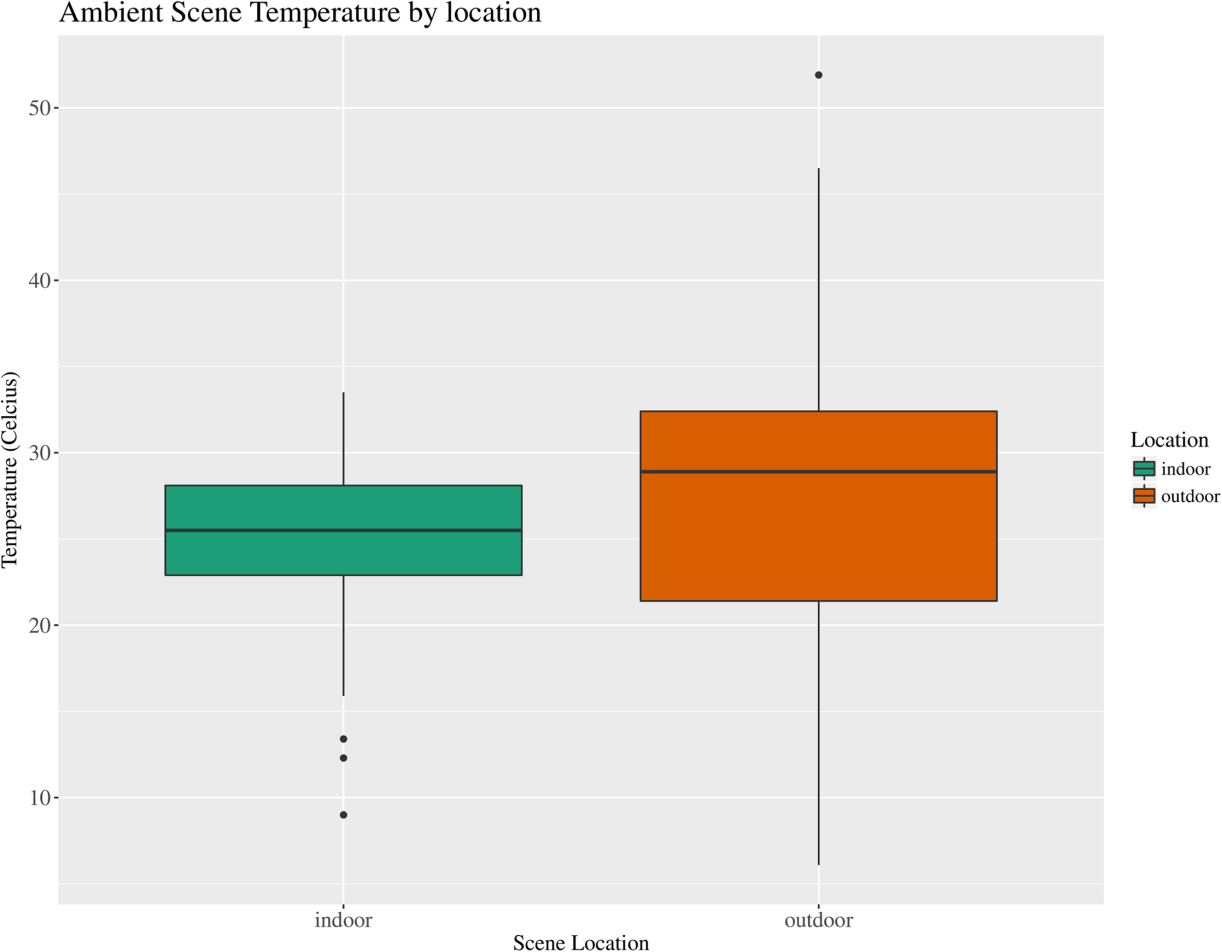
Boxplot of median ambient scene temperature (°C) by scene location. Boxplot illustrating the median ambient air temperature (°C) and associated variation recorded near the decedent at scenes located indoors and outdoors for forensic entomology cases over the study period (January 2013–April 2016). (N = 192).

Perhaps the most important thing to consider when examining these data is that they represent casework and not the results of a planned ecological study, which imposes certain limitations on the statistical analyses completed and the conclusions drawn. Of the 203 cases analyzed, six were from hospitals without an associated scene investigation (3.0%), 65 were from outdoor scenes (32.0%) and 132 were from indoor scenes (65.0%). Scene type appears to play a significant role in not only the species of insects encountered (Table 2) but in the conditions one may encounter during scene investigation (Table 1; Figs 1 and 2). It is also important to note that the scene conditions reported here do not necessarily represent the conditions experienced by the colonizing insects because these data were collected when insects were collected and conditions at the scene may constantly change as decomposition progresses and as scene investigation is conducted. These data represent the conditions in which these insects might be found during the course of routine death investigations, which may be valuable to understanding the conditions under which insects colonize human remains; a goal of applied forensic entomology as it is used in casework.

### Scene types

#### Hospitals

Insect analysis involving hospital cases typically involved the identification of insects or insect artifacts such as honeybee (*Apis mellifera* Linneaus; Hymenoptera: Apidae) stingers (Table 2) rather than TOC estimation. In those cases where a TOC estimate was generated, the cases involved peri-mortem myiasis wherein the decedent was colonized by insects, namely flies (Diptera; Table 2), at some time point before arrival to the emergency department at the hospital and before death. Estimation of TOC for decedents that come from the hospital with fly larvae allows for the determination of colonization that can account for myiasis. Otherwise without any extenuating circumstances, such as a significant discrepancy between the stage of the insect larvae and the stage of decomposition or known medical history, myiasis can be overlooked [22]. Even when myiasis is known the calculation of the TOC estimate can be complicated by unknown factors related to which temperatures to use when calculating the accumulated degree hours (ADH) required for development [22]. This can be due to health related factors such as reduced circulation to extremities or due to unknown timing of demise and the switch from the use of body temperature to ambient temperature in calculations [30].

Thus far, the insects and arthropods associated with decedent’s coming from the hospital have included honeybee, typical human parasites including lice and ticks, and blow flies (Table 2). There is a growing appreciation for the information that these organisms can provide. As an example, a recent case demonstrated the potential of ticks to be useful to unraveling the travel history and potentially the next of kin (NOK) information for a decedent. In this case, the decedent came from the hospital emergency room with very little information, except that he had been traveling and had been dropped off at the hospital by a friend. The decedent had a partially engorged tick attached to his right ear that detached upon his arrival to the morgue and was quickly collected. This particular specimen was identified as a nymphal *Amblyomma* sp. (Ixodida: Ixodidae) most likely *A*. *cajennense* Fabricius or *A*. *imitator* Kohls, which both have a known distribution in South Texas, Mexico and Central America [31]. Once all the investigative information was put together, the range for the tick and the decedent’s travel history matched. One can imagine in the absence of identification and/or NOK information that notice could be sent to consulate offices in the range of the tick to help in the search or for a decedent that is from within the United States to law enforcement agencies in the range of the tick species. One could also use the tick to test for tick-borne disease, potentially for surveillance or in determination of cause of death. Insects and other arthropods have the potential to help with a variety of other types of questions related to a decedent’s life and death; therefore, collection of specimens, no matter how trivial, can sometimes prove to be incredibly useful to the investigation.

#### Outdoor scenes

Cases involving specimens from decedents found outdoors primarily involve estimating TOC for the purposes of estimating PMI. This is the classic scenario in forensic entomology from which many of the protocols and standards were derived [18,32]. Fig 1 illustrates the official manner of death classifications for HCIFS forensic entomology cases from January 2013 through April 2016. It shows that outdoor scenes (71) are outnumbered by those that occur indoors (132). A majority (98) of all of the cases involve natural manner of death classifications as the official manner of death. Initially these cases come into the morgue as undetermined cases due to the difficulty of assessing trauma of decomposing decedents. The number of cases involving undetermined, accident and suicide manners of death that involve insects and occur outdoors are approximately equal to those that occur indoors. Noticeably, there are a higher percentage of homicide cases (12.3%, Fig 1) involving insects from decedents at scenes located outdoors than indoors. This is reflected in the initial development of forensic entomology tools for the outdoor scene as the initial opportunities of forensic entomology casework were to assist with homicide investigations [1]. It follows that decomposing decedents have fewer delays in colonization related to insect access and more opportunities for temperature modifications that may accelerate decomposition (e.g. direct sun exposure).

Analysis of factors associated with outdoor scenes in Harris County involves significantly higher average temperature (28.0°C; Table 3; Fig 2) but few other significant differences in the other factors observed when compared to indoor scenes (Table 3). The median for TOC estimates from outdoor scenes was slightly shorter but not significantly different from those estimated from indoor scenes (S2 Fig). This result makes logical sense as higher temperatures lead to shorter estimates of TOC (quicker accumulation of accumulated degree hours) which may lead toward a tendency to underestimate TOC. The opposite effect may occur at indoor scenes were cooler temperatures lead to longer estimates of TOC (Fig 2). Differences in temperature may also be a factor related to differential insect presence at indoor and outdoor scenes.

**Table 3:** Kruskal-Wallis chi-squared test results.

Comparison	Kruskal-Wallis Χ^2^ Test Statistic	d.f.	P-value
Ambient scene temperature by scene location^1^	6.8397	1	0.008916*
Relative humidity by scene location^1^	2.7817	1	0.09534
Length of TOC estimate by scene location	3.6595	2	0.05575
Ambient scene temperature by stage of decomposition	1.9573	4	0.7436
Ambient scene temperature by Manner of death	11.708	4	0.01966*
Relative humidity by Manner of death	13.867	4	0.00773*
Length of TOC estimate by Manner of death	25.338	4	>0.0001*
Scene relative humidity by stage of decomposition	3.0391	4	0.5513
Length of TOC estimate by stage of decomposition	71.849	4	>0.0001*

Kruskal-Wallis Chi-Squared statistical comparisons for natural log transformed data for ambient scene temperature, relative humidity and length of the time of colonization (TOC) estimate (days) by scene location, stage of decomposition and Manner of death.

^1^Temperature and relative humidity comparisons did not include hospital cases, as these measurements were not made at the hospital.

*indicates a significant difference at the α = 0.05 level.

Only one insect species was significantly associated with being encountered at outdoor scenes, the green bottle fly, *Lucilia eximia* (Wiedemann) (Diptera: Calliphoridae) (Table 4). All other insect groups, which were encountered in five or more cases, were either found to have a significantly higher probability to be encountered at indoor scenes or to be encountered at both indoor and outdoor scenes (Table 4). According to the binomial test, the probability of collecting *L*. *eximia* indoors is significantly lower than at an outdoor scene based on the data currently available in Harris County (Table 4). This suggests that finding this species on a decedent endorses outdoor colonization of the decedent when considered alone. However, three cases involved the collection of *L*. *eximia* at indoor scenes (Table 2) suggesting that under some circumstances this species does colonize bodies located indoors. These exception cases included a double homicide where both decedents were located near the door in a closed residential garage and a natural case where fly access may have been gained the attached garage. The combination of species encountered on a decedent may suggest movement of the decedent if for example, when *L*. *eximia* is found together with a strongly indoor colonizing species like *Megaselia scalaris* Loew (Diptera: Phoridae) (Table 4), but this may only be of importance when other scene indicators suggest this possibility. The opposite scenario may also be suggested if a strongly indoor colonizing species such as *M*. *scalaris* is found on a decedent located outdoors. In the casework analyzed here, it was actually uncommon for bodies to be found to be colonized by a single insect species with only 14.2% (28 of 197) of cases found to have only a single colonizing species and the rest with more than one. Further study of species combinations and the location of the decedent will be needed to parse out the scenarios where body movement may be indicated by species presence/absence on the body.

**Table 4:** Binomial test results suggesting indoor or outdoor scene collection.

Groups with >/ = 5 cases	N	Probability of success	95% confidence interval of success		P-value
			min	max	
*Blaesoxipha plinthopyga*	39	0.9744	0.8652	0.9994	1.46E-10*
*Megaselia scalaris*	67	0.9701	0.8963	0.9964	<2.20E-16*
*Lucilia cuprina*	11	0.9091	0.5872	0.9977	0.01172*
Sarcophagidae	103	0.9029	0.8287	0.9525	<2.20E-16*
*Synthesiomyia nudiseta*	18	0.8889	0.6529	0.9862	0.001312*
*Musca domestica*	5	0.8000	0.2836	0.9949	0.375
*Phormia regina*	31	0.5806	0.3908	0.7545	0.4731
Calliphoridae	134	0.5448	0.4565	0.6310	0.342
*Chrysomya rufifacies*	78	0.5000	0.3846	0.6154	1
*Cochliomyia macellaria*	46	0.4783	0.3289	0.6305	0.883
*Hydrotea* sp.	21	0.4762	0.2571	0.7022	1
*Dermestes maculatus*	22	0.4091	0.2071	0.6365	0.5235
*Piophila casei*	7	0.2857	0.0367	0.7096	0.4531
*Hermetia illucens*	8	0.2500	0.0319	0.6509	0.2891
*Lucilia eximia*	22	0.0000	0.0000	0.1544	4.77E-07*
Nitidulidae	5	0.0000	0.0000	0.5218	0.0625

Exact binomial test results indicating the probability of success as defined by encountering a particular insect group indoors. The 95% confidence interval and P-value for each test are also provided. Statistics were only calculated for cases where each insect group was encountered five or more times during the study period.

*indicates a significant difference at the α = 0.05 level.

#### Indoor scenes

Cases involving insects collected indoors also have a primary goal of TOC estimation for the purpose of estimating PMI. A majority of the forensic entomology case reports from Harris County over the analyzed period were from indoor scenes and they encompassed every manner of death classification except fetal (Fig 1). These data suggest expanding forensic entomology tools and research to understanding insect colonization indoors, which is an area of forensic entomology that has received comparatively less attention [33,34]. The average ambient scene temperature observed at indoor scenes (25.2°C) was significantly lower than that observed at outdoor scenes (Table 3; Fig 2). As mentioned earlier the number of cases that involve manner of death classifications involving accidents, suicides and undetermined classifications involve scenes located indoors and outdoors suggesting a need for more tools for indoor scenes. Indoor scenes can be complicated by multiple factors that affect insect colonization (e.g. delays in colonization; [33]), temperature modification (e.g. air conditioning, heating), co-located decomposing remains (e.g. decomposing pets; [35]), 24-hour lighting which may allow for nocturnal oviposition (e.g. a TV or lamp left on at the scene;[36]), other sources of insects in the residence that may reduce or eliminate a delay in colonization (e.g. hoarding of trash and food) and a number of other case specific factors that may affect the TOC estimate. Many of these complicating factors have as yet unknown effects on TOC estimation but have the potential to have significant impacts on PMI estimation.

Another significant observation regarding indoor scenes is in the different insects found at indoor scenes. Just as some insect species appear to have preference for outdoor scenes there appears to be a strong association between indoor scenes and several insect groups including *Megaselia scalaris*, Sarcophagidae (Diptera) including *Blaesoxipha plinthopyga* (Wiedemann) (Diptera: Sarcophagidae), *Synthesiomyia nudiseta* Wulp (Diptera: Muscidae) and *Lucilia cuprina* (Wiedemann) (Diptera: Calliphoridae) as can be observed in the binomial test statistics presented in Table 4. This suggests when each of these species, considered alone, may represent an indicator of indoor colonization or at the very least allow for the consideration of an indoor scene from the insect’s perspective. A car in a covered carport or a storage container where a decedent was living may not be what may typically be considered indoor scenes but from the insect’s perspective, they may fulfill colonization preferences. Therefore, scene indicators and context should all be taken into account when considering insect specimens as indicators of body movement.

These results are similar to those observed by other researchers in other geographic locations. On the Hawaiian Island of Oahu, Goff [37] found that three species were strong indicators of indoor scenes including a species of the Phoridae *Megaselia scalaris*, the Sarcophagidae *Bercaea haemorrhoidalis* (Fallen) and the Muscidae *Stomoxys calcitrans* Linneaus. While not exactly the same species encountered in the current study, a similar combination of representatives of the same fly families was found with the same Phoridae *M*. *scalaris*, the Sarcophagidae *B*. *plinthopyga* and the Muscidae *S*. *nudiseta* indicating indoor colonization. Goff also found that there was a wider diversity of fly species indoors, during the earlier phases of decomposition. In the cases analyzed in the current study there was a similar, though small, result with an average of 1.9 (+/-1.3 standard deviation) species of flies found from outdoor scenes and an average of 2.4 (+/-1.3 standard deviation) fly species encountered in specimens from outdoor scenes overall. The Hawaiian study also reported a higher diversity of beetles encountered outdoors and this was also a trend that was observed in the current study although beetles were more rarely collected (Table 2). In Western Canada Anderson [33] found that most blow fly species colonizing experimentally placed pigs indoors and outdoors overlapped with several species appearing to be restricted to the outdoors. Similar results were obtained in the current study where the only species that was strongly associated with outdoor scenes was *L*. *eximia* (Tables 2 and 4). However, in an analysis of casework Anderson [14] found that in British Colombia, Sarcophagidae and Phoridae did not appear to be significant colonizers and did not appear to have the same dichotomy based on scene location. When one considers the climate similarities and differences among the three geographic locations, such as the temperatures, relative humidity (Table 1) and precipitation encountered at scenes, Harris County along the Gulf Coast is perhaps more similar to those encountered in Hawaii rather than British Columbia. Furthermore, climate change may lead to changes in species ranges over time [38,39] or to differentiation in developmental progression in different populations of the same species. This highlights the necessity and importance of collecting and publishing these data for different geographic locations, different climatic regions and different species assemblages rather than relying only on a handful of studies.

Some species of insects and arthropods analyzed in Harris County casework have only been found indoors thus far. The flesh fly *Blaesoxipha plinthopyga* is the only fly, which has been identified exclusively indoors. This fly was not known to be a forensically significant fly in the US until quite recently [40] and this is perhaps due in part to the difficulty in identification of Sarcophagidae, which requires either prepared adult male flies [41] and expert morphological expertise associated with male genitalia or expertise in the molecular systematics of Sarcophagidae [42,43] and adequate laboratory capacity. Therefore, Sarcophagidae have often been relegated to the family level without further identification. Nevertheless, even when identification is possible there are significant gaps in the literature for detailed development datasets for many of the forensically important Sarcophagidae species. The mite, *Myianoetus muscuarum* Linneaus (Acariformes: Histiostomatidae), has only been found indoors but it has only been found in few cases thus far ([44] and Table 2) and it may be the case that the more it is looked for the more it may be found. The other species that have been found exclusively indoors have very low case numbers and may have just not been collected frequently enough to establish habitat or colonization preferences for these insects/arthropods (Table 2).

### Insects/Arthropods

The introduction of routine forensic entomology into the Medical Examiner’s office has allowed for the observation of several new associations between insects, arthropods and human decomposition. One of those observations was the first report of what was determined to be myiasis of a decedent shortly before his death by *L*. *eximia* and *Chrysomya rufifacies* (Macquart) (Diptera: Calliphoridae) for the first time on a human in the US [30]. In another case, the blue blow fly, *Calliphora coloradensis* Hough (Diptera: Calliphoridae) was collected from an outdoor scene in February 2014, associated with a decedent in an advanced state of decomposition and vertebrate scavenging. The specimen was reared from a pupa located next to the decedent and it was the first time that this species had been collected from a decedent. It is known to occur in Texas [11] but has not to the author’s knowledge, been collected in association with a decomposing human in Harris County. It has been previously recorded as an adult associated with a human corpse found at high elevation in Colorado but was not observed as a colonizer of the remains [45]. In another case the tawny crazy ant, *Nylanderia fulva* (Mayr) (Hymenoptera: Formicidae) was recorded on a decomposing human body in Harris County for what appears to be the first time (Table 2). However, these species may be regularly observed at scenes but not regularly collected and reported upon because they are not typically related to time of colonization estimation.

Aside from new insect associations, other new arthropod associations are being made as well. The mite, *M muscuarum* was recently established to have a relationship with *S*. *nudiseta* at indoor scenes [44] and has been found in additional cases since this publication was completed. Mites continue to be encountered at scenes and with help of Dr. B. OConnor (University of Michigan), identifications of the mite species are being made in order to develop a taxonomic index and to be able to associate the mite fauna with timing in the decomposition process for this geographic location. A nematode or nematodes species were recently found in association with decomposing decedents during the rearing of Calliphoridae and Stratiomyidae specimens and their identity is being investigated by Dr. Y. Qing (Canadian National Collection of Nematodes).

The location of Harris county being on the Gulf Coast and near the southern US border presents the opportunity to make observations related to new species introductions as well as documenting new associations. The forensic entomologist can therefore serve in a capacity to identify foreign species introductions and to notify relevant interested parties. This applies to not only those insects that are used for TOC estimation but also those that may be relevant to public health and cause of death determination as new arthropod-borne diseases are introduced to the US.

### Seasonality

The seasonality of the blow flies encountered in Harris County follows patterns generally accepted for Calliphoridae in North America [46]. The typical cool weather blue bottle flies, *Calliphora* spp. are found in the winter months and other species such as the black blow fly, *Phormia regina* Robineau-Desvoidy (Diptera: Calliphoridae), and the Muscidae species, *S*. *nudiseta* are active in the spring months (Table 2). The green/bronze bottle flies, *Lucilia* spp. are most often collected in the hot summer months but can be encountered throughout the spring through the fall months (Table 2). The introduced species, the hairy maggot blow fly, *Chrysomya rufifacies*, and the oriental latrine fly, *Chrysomya megacephala* Fabricius (Diptera: Calliphoridae), appear to be active year-round in some years whereas in other years they can be absent in the winter months (Table 2). It is a very rare occurrence for there to be snow in Harris County and temperatures below 0.0°C rarely persist for more than a few days if at all [47]. These climate characteristics probably help the introduced tropical Calliphoridae species persist through the winter as freezing temperatures are thought to restrict their distribution in North America [48].

The Sarcophagidae species, *B*. *plinthopyga*, and the Phoridae species, *M*. *scalaris*, are not as seasonally restricted and appear to be collected in casework year-round (Table 2). It would seem that this might be partially explained by their exploitation of indoor scenes. Currently no data is available on the dispersal patterns of either of these fly species. Other Phoridae species appear to be capable of transportation via wind and human modes of transport [49]. Preliminary data suggests population structure exists within *M*. *scalaris* populations in the US even across relatively small geographic distances [50]. Indoor environments with their cooler and less variable temperature range (Fig 2) may offer refuge when outdoor conditions are unfavorable allowing populations to persist locally during bad weather.

### Manner of death

**Fig 3:**
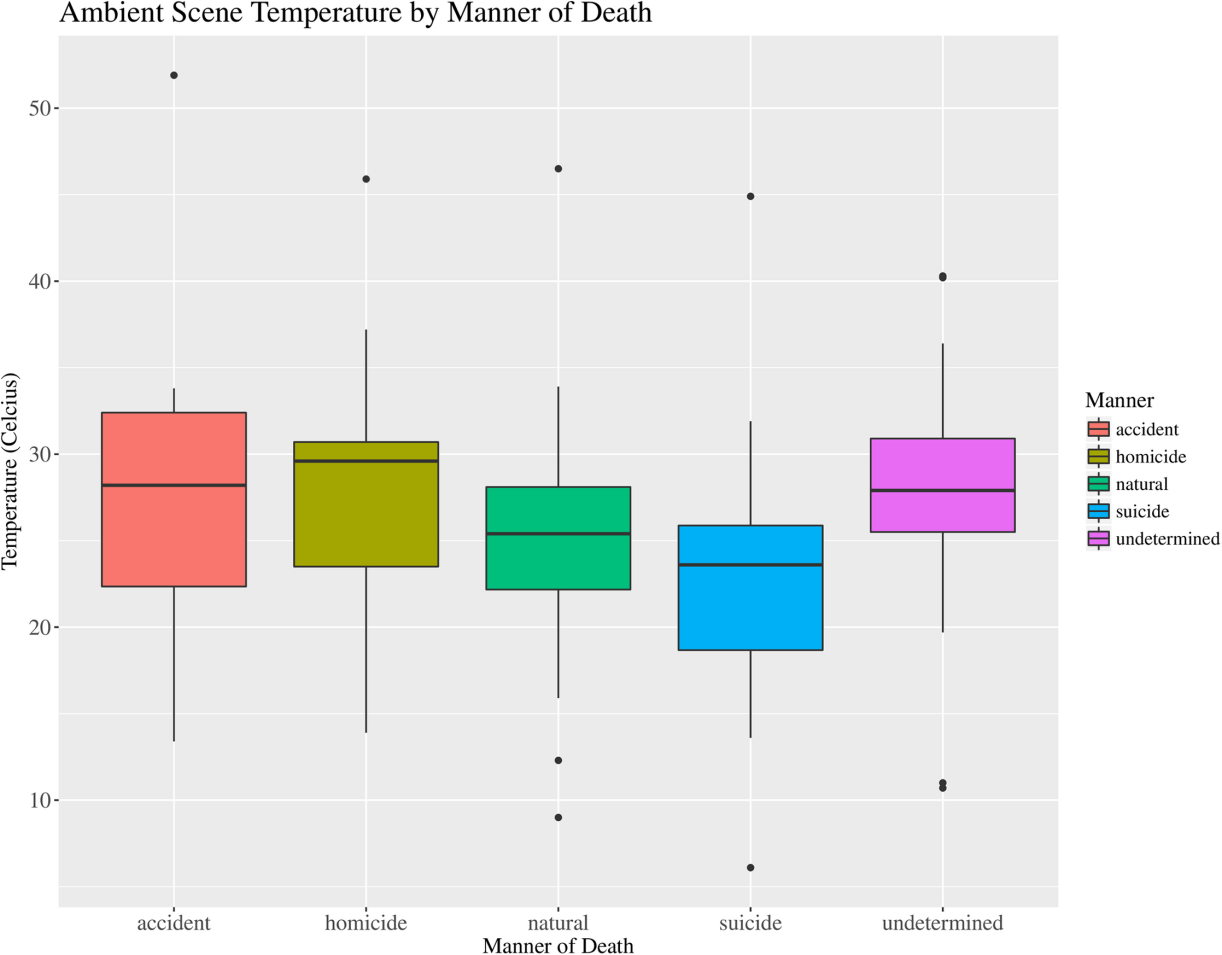
Boxplot of median ambient scene temperature (°C) by manner of death. Boxplot illustrating the median ambient air temperature (°C) and associated variation recorded near the decedent at scenes by the Manner of death classification for forensic entomology cases over the study period (January 2013–April 2016). (N = 192).

**Fig 4:**
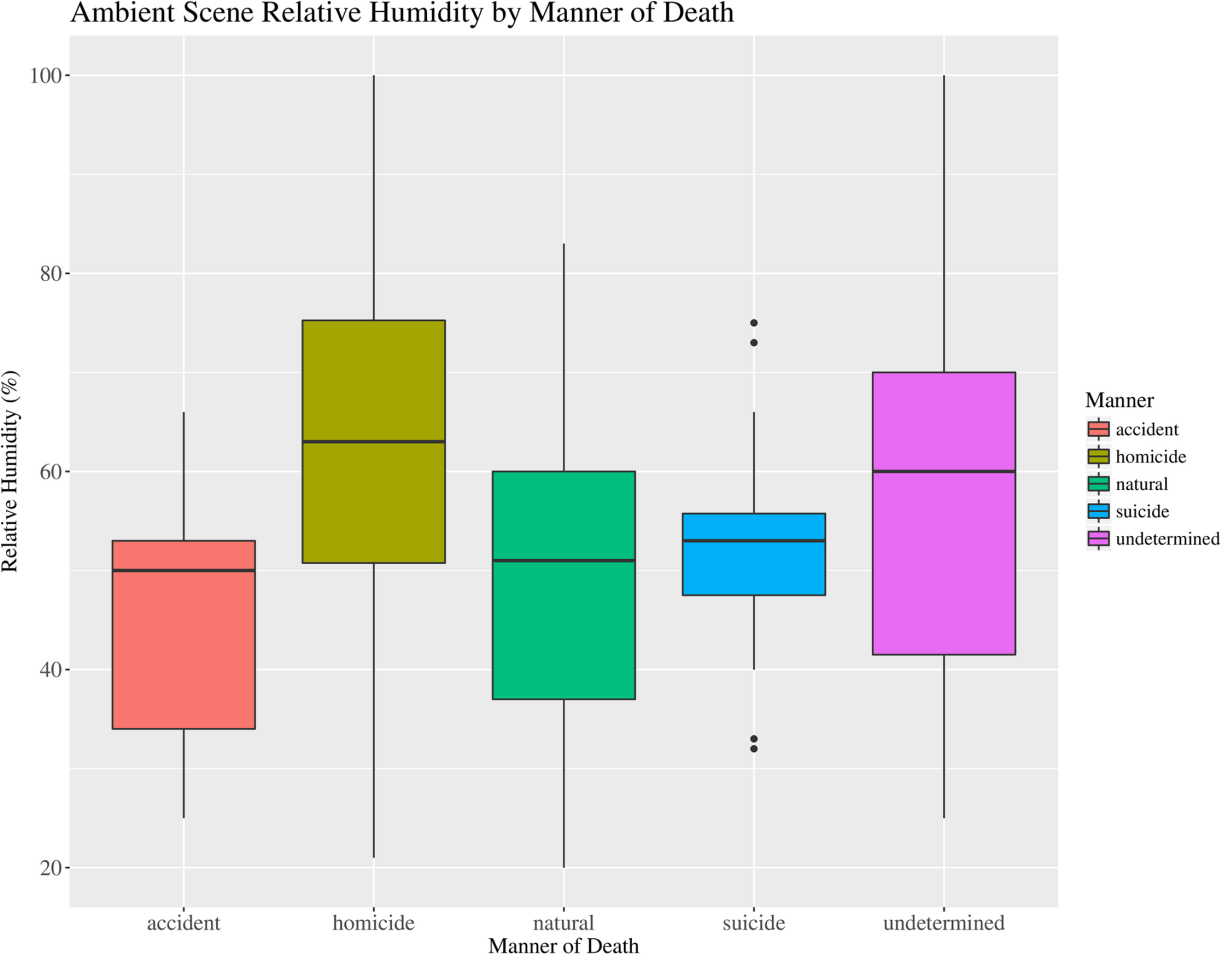
Boxplot of median relative humidity (%) by manner of death. Boxplot illustrating the median relative humidity (%) and associated variation recorded near the decedent at scenes by the Manner of death classification for forensic entomology cases over the study period (January 2013–April 2016). (N = 192).

**Fig 5:**
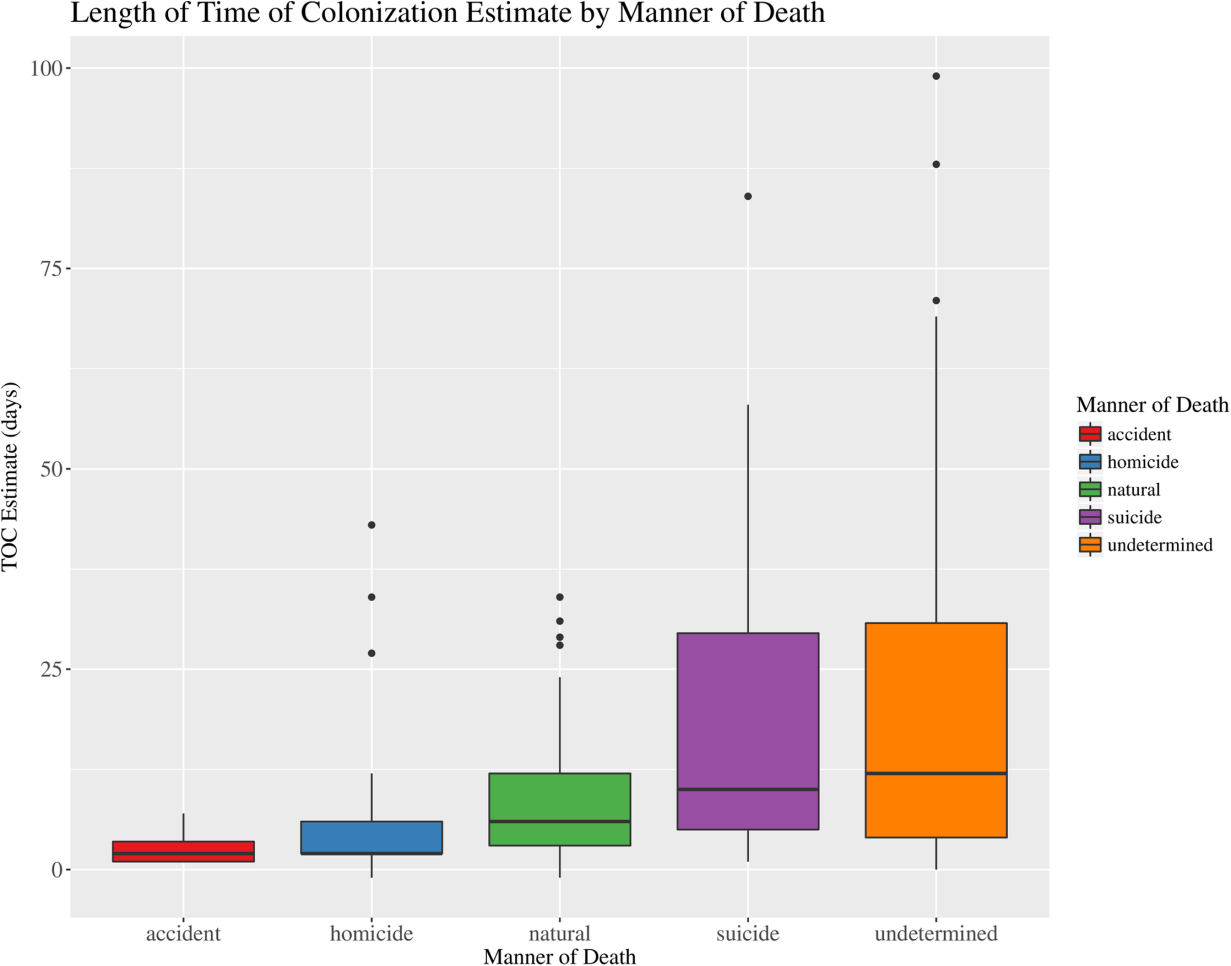
Boxplot of median time of colonization (TOC) estimate (days) by manner of death. Boxplot illustrating the median time of colonization (TOC) estimate (days) and associated variation for decedents by the Manner of death classification for forensic entomology cases over the study period (January 2013–April 2016). (N = 192).

The results of the Kruskal-Wallis Chi-squared analysis indicate significant differences (Table 3) in the ambient scene temperature (Fig 3), observed scene relative humidity (Fig 4) and length of the TOC estimate (Fig 5). The significance of these factors is most likely related to correlation with other data. For example the higher proportion of homicides from outdoor scenes would be expected to have a higher ambient and development temperatures and hence shorter TOC estimates. Another possible correlation is related to undetermined cases, which tend to include cases of advanced and skeletal remains that have been exposed for long periods of time and have long TOC estimates. Therefore, the importance of the significant differences observed for Manner of death seems low.

### Stage of decomposition

**Fig 6:**
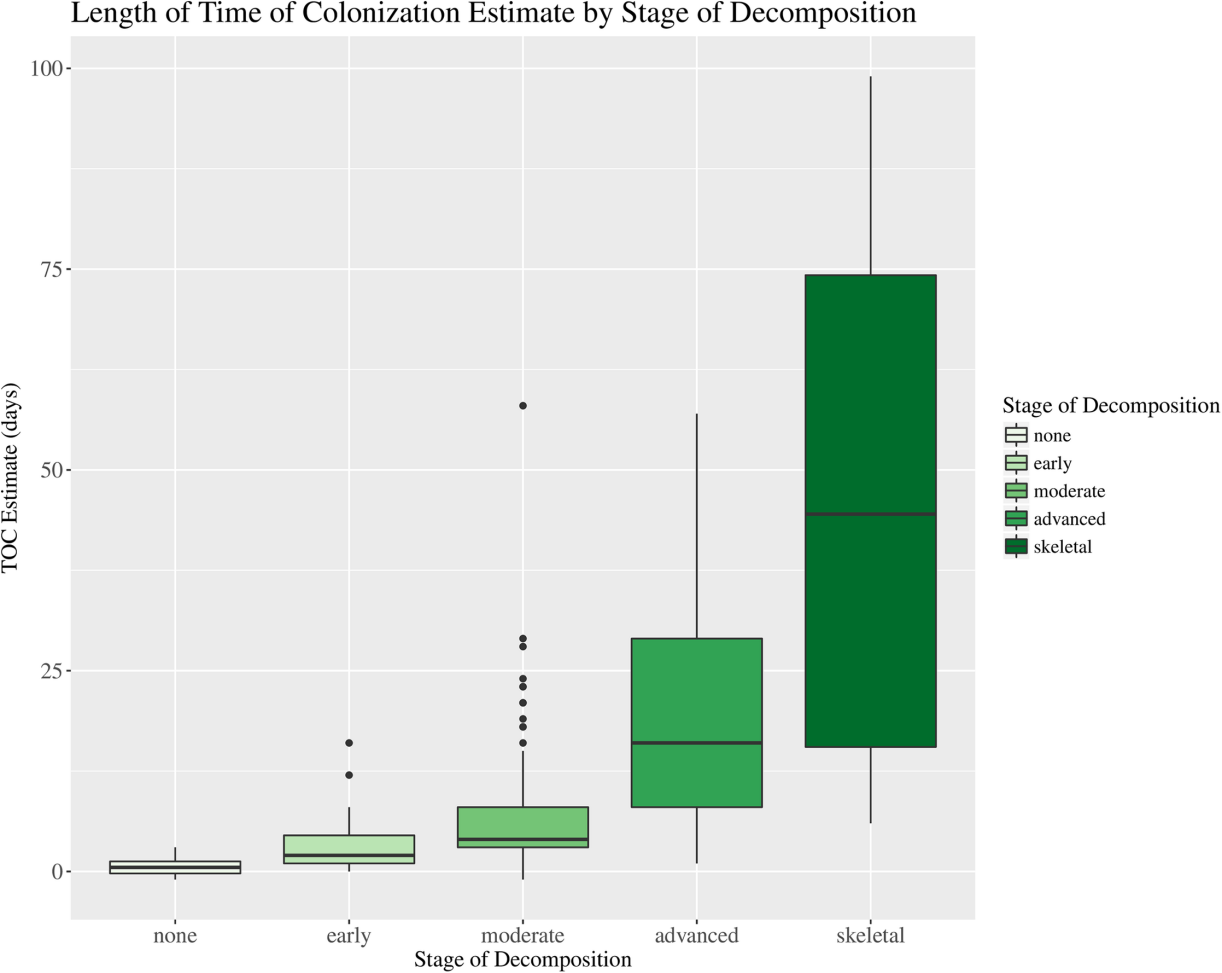
Boxplot of median time of colonization (TOC) estimate (days) by stage of decomposition. Boxplot illustrating the median time of colonization (TOC) estimate (days) and associated variation for decedents found in different stages of decomposition for forensic entomology cases over the study period (January 2013–April 2016). (N = 192).

A significant Kruskal-Wallis Chi-squared result was also indicated for length of the TOC estimate by stage of decomposition (Table 3, Fig 6). This result seems intuitive such that the greater the level of decomposition the longer the individual has been deceased and the longer the TOC estimate generated by examination of the associated insects. As can also be seen in Fig 6, the greater the stage of decomposition the higher level of variability observed among estimates.

## Conclusions

The integration of full time forensic entomology services to the medical examiner’s office in Harris County has revealed new observations, confirmed long-standing hypotheses and opened up a wide range of new opportunities. The casework described here is not an endpoint and new species observations and associations continue to be revealed. This study represents a reference point from which to describe additional observations and trends and from which to compare other geographic locations and species assemblages. These data also remind us that depending upon generalizations and assumptions can be misleading and that keeping an open mind and collecting something that may at first seem trivial may prove to be exceptionally useful.

The data presented here also have illustrated several persistent issues in the application of forensic entomology to casework. While molecular identification tools are being more widely developed and applied to morphologically difficult groups (e.g. Sarcophagidae and Muscidae) there are significant gaps in development datasets. A single development dataset is available for *B*. *plinthopyga* which was never intended for use in application to forensic entomology as it is includes total generation time [51]. Yet this species is considerably important to casework in Harris County (Table 2). There are no known published development datasets for *C*. *coloradensis*, *Calliphora livida* Hall (Diptera: Calliphoridae) or *Dermestes maculatus* DeGeer (Coleoptera: Dermestidae) using a diet that approximates human tissue (i.e. human or porcine tissue [52]). The suggested practice of using closely related species to approximate development for those species that have missing data introduces unnecessary uncertainty in an estimate of TOC and its application to PMI. Furthermore there is growing awareness that different populations of the same species have different developmental responses to the same temperatures over large [53] and relatively small geographic distances [54]. Several of the species that are common and important to casework in Harris County lack local development datasets including *C*. *megacephala*, *S*. *nudiseta*, *L*. *eximia* and *L*. *cuprina*. This strongly suggests that using development datasets from populations not local to the collection location for the case may introduce uncertainty to a case that becomes difficult to incorporate into a TOC estimate.

While missing development datasets can be generated, other issues continue to haunt forensic entomology casework that are not and will not be as easy to remedy. In 1992, Catts [55] outlined several complications in using insects to estimate postmortem interval. These issues, such as maggot mass heat, insect access and entomotoxicology, continue to be sources of uncertainty for the application of forensic entomology to casework. It has only been relatively recently that there has been an increased awareness for the need to account for uncertainty and sources of error in forensic entomology within the context of all forensic sciences in the US [56]. In addition, while identifying potential sources of error is more widely appreciated, accounting for how these errors impact TOC and PMI estimates has not yet been resolved. Tackling these challenges will require strong collaboration between academic and practitioner parts of the community, and the results will make forensic entomology an even more powerful tool in understanding the post-mortem interval.

## Supporting information

S1 Table Frequently used taxonomic keys. Some of the most frequently used taxonomic keys and literature for forensic entomology casework in Harris County, Texas, USA from January 2013 through April 2016.
(DOCX)

S1 File Normality test results for transformed data. Results of normality tests of the transformed temperature, relative humidity and estimated post-mortem interval data. Includes Q-Q plots of the data.
(PDF)

S2 File Kruskal-Wallis test results for transformed data. The results of the Kruskal-Wallis chi-squared tests on the transformed temperature, relative humidity and estimated post-mortem interval data.
(PDF)

S3 File De-identified spreadsheet of case data. Spreadsheet containing de-identified case data for insects collected at scenes in Harris County, Texas, USA from January 2013 through April 2016.
(XLSX)

S1 Fig Boxplot of relative humidity by scene location. Boxplot illustrating the median and range of observed relative humidity for indoor and outdoor scene locations.
(TIF)

S2 Fig Boxplot of estimated length of time of colonization by scene location. Boxplot illustrating the median and range of the length of time of colonization estimates (days) by scene location (indoors vs. outdoors).
(TIF)

S3 Fig Boxplot of observed ambient scene temperature by stage of decomposition. Boxplot illustrating the median and range of the observed ambient scene temperatures (°C) by the observed stage of decomposition for the decedent.
(TIF)

S4 Fig Boxplot of observed relative humidity by stage of decomposition. Boxplot illustrating the median and range of observed relative humidity at scene by the observed stage of decomposition for the decedent.
(TIF)

## References

[1] Catts EP, Goff ML. Forensic entomology in criminal investigations. Annu Rev Entomol. 1992;37: 253–272. doi: 10.1146/annurev.en.37.010192.001345 1539937 10.1146/annurev.en.37.010192.001345

[2] Byrd JH, Castner JL. Forensic Entomology: The Utility of Arthropods in Legal Investigations, Second Edition 2nd ed. Byrd JH, Castner JL, editors. Boca Raton, FL: Taylor & Francis; 2009 doi: 10.4001/003.018.0221

[3] Hwang C, Turner BD, Monitoring D, County C. Spatial and temporal variability of necrophagous Diptera from urban to rural areas. Med Vet Entomol. 2005;19: 379–391. doi: 10.1111/j.1365-2915.2005.00583.x 16336303 10.1111/j.1365-2915.2005.00583.x

[4] Carvalho LML, Thyssen PJ, Linhares AX, Palhares FAB. A Checklist of Arthropods Associated with Pig Carrion and Human Corpses in Southeastern Brazil. Mem Inst Oswaldo Cruz. 2000;95: 135–138. doi: 10.1590/S0074-02762000000100023 10656720 10.1590/s0074-02762000000100023

[5] Tenorio FM, Olson JK, Coates CJ. Decomposition studies, with a catalog and descriptions of forensically important blow flies (Diptera: Calliphoridae) in Central Texas. Southwest Entomol. 2003;28: 267–272.

[6] Goddard J. Blow fly bait preferences and seasonal activity in Bexar County, Texas. Southwest Entomol. 1988;13: 131–135.

[7] Snow JW, Coppedge JR, Broce AB, Goodenough JL, Brown HE. Swormlure: Development and use in detection and suppression systems for adult screwworm (Diptera: Calliphoridae). Bull Entomol Soc Am. 1982;28: 277–284. doi: 10.1093/besa/28.3.277

[8] Coppedge JR, Ahrens EH, Snow JW. Swormlure-2 baited traps for detection of native screwworm flies. J Econ Entomol. 1978;71: 573–575. doi: 10.1093/jee/71.4.573

[9] Richard RD, Ahrens EH. New distribution record for the recently introduced blow fly *Chrysomya rufifacies* (Macquart) in North America. Southwest Entomol. 1983;8: 216–218.

[10] Wells JD, Greenberg B. Resource use by an introduced and native carrion flies. Oecologia. 1994;99: 181–187. doi: 10.1007/BF00317099 28313964 10.1007/BF00317099

[11] Wells JD, Greenberg B. Effect of the Red Imported Fire Ant (Hymenoptera Formicidae) and carcass type on the daily occurence of postfeeding carrion-fly larvae (Diptera: Calliphoridae, Sarcophagidae). J Med Entomol. 1994;31: 171–174. doi: 10.1093/jmedent/31.1.171 8158622 10.1093/jmedent/31.1.171

[12] Early M, Goff ML. Arthropod succession patterns in exposed carrion on the island of Oahu, Hawaiian Islands, USA. J Med Entomol. 1986;23: 520–531. doi: 10.1093/jmedent/23.5.520 3772956 10.1093/jmedent/23.5.520

[13] Goff ML, Early M, Odom CB, Tullis K. A preliminary checklist of arthropods associated with exposed carrion in the Hawaiian Islands. Proc Hawaiian Entomol Soc. 1986;26: 53–57. Available: http://hdl.handle.net/10125/11192

[14] Anderson GS. The use of insects in death investigations: an analysis of cases in British Columbia over a five year period. J Can Soc Forensic Sci. 1995;28: 277–292. doi: 10.1080/00085030

[15] Chapter 49. Inquests upon dead bodies. Title 1 Code of Criminal Procedure. State of Texas; 1965. Available: http://www.statutes.legis.state.tx.us/Docs/CR/htm/CR.49.htm

[16] Haskell NH, Williams RE. Entomology & Death: A procedural guide 2nd ed. Clemson, SC: Joyce’s Print Shop; 2008.

[17] Amendt J, Campobasso CP, Gaudry E, Reiter C, LeBlanc HN, Hall MJR. Best practice in forensic entomology—standards and guidelines. Int J Legal Med. 2007;121: 90–104. doi: 10.1007/s00414-006-0086-x 16633812 10.1007/s00414-006-0086-x

[18] Byrd JH, Lord WD, Wallace JR, Tomberlin JK. Collection of entomological evidence during legal investigations In: Byrd JH, Castner JL, editors. Forensic Entomology: The Utility of Arthropods in Legal Investigations. Second Boca Raton, FL: CRC Press; 2010 pp. 127–175.

[19] Byrd JH, Tomberlin JK. Laboratory Rearing of Forensic Insects In: Byrd JH, Castner JL, editors. Forensic Entomology: The Utility of Arthropods in Legal Investigations. 2nd ed. Boca Raton, FL: CRC Press; 2010 pp. 177–200.

[20] Adams Z, Hall MJR. Methods used for the killing and preservation of blowfly larvae, and their effect on post-mortem larval length. Forensic Sci Int. 2003;138: 50–61. doi: 10.1016/j.forsciint.2003.08.010 14642719 10.1016/j.forsciint.2003.08.010

[21] Google Inc. Google Earth Pro [Internet]. Google, Inc; 2015. Available: https://www.google.com/earth

[22] Wells JD, Lamotte LR. Estimating the postmortem interval In: Byrd JH, Castner JL, editors. Forensic Entomology: The Utility of Arthropods in Legal Investigations. Second Boca Raton, FL: CRC Press; 2010 pp. 367–388.

[23] Fox J. The R Commander. A Basic Statistics Graphical User Interface to R. J Stat Softw. 2005;14: 1–42. Available: http://www.rcommander.com/

[24] McDonald JH. Handbook of Biological Statistics [Internet]. Baltimore, MD: Sparky House Publishing; 2008 Available: http://udel.edu/~mcdonald/statintro.html

[25] R Core Team. R: A language and environment for statistical computing [Internet]. Vienna, Austria; 2017. Available: https://www.r-project.org

[26] RStudio Team. RStudio: Integrated Development for R [Internet]. Boston, MA: RStudio, Inc.; 2015. Available: http://www.rstudio.com

[27] Wickham H. ggplot2: Elegant Graphics for Data Analysis [Internet]. New York, NY: Springer-Verlag; 2009 Available: http://ggplot2.org

[28] Neuwirth E. Package “RColorBrewer” [Internet]. 2014. Available: https://cran.r-project.org/web/packages/RColorBrewer/index.html

[29] Chang W. extrafont: Tools for using fonts [Internet]. 2014. Available: https://github.com/wch/extrafont

[30] Sanford MR, Whitworth TL, Phatak DR. Human wound colonization by *Lucilia eximia* and *Chrysomya rufifacies* (Diptera: Calliphoridae). Myiasis, perimortem or postmortem colonization? J Med Entomol. The Oxford University Press; 2014;51: 716–719. doi: 10.1603/ME13229 24897868 10.1603/me13229

[31] Keirans JE, Durden LA. Illustrated key to nymphs of the tick genus Amblyomma (Acari: Ixodidae) found in the United States. J Med Entomol. 1998;35: 489–495. doi: 10.1093/jmedent/35.4.489 9701933 10.1093/jmedent/35.4.489

[32] Haskell NH, Williams RE. Collection of entomological evidence at the death scene In: Catts EP, Haskell NH, editors. Entomology & Death: A Procedural Guide. Clemson, SC: Joyce’s Print Shop; 1990 pp. 82–97.

[33] Anderson GS. Comparison of decomposition rates and faunal colonization of carrion in indoor and outdoor environments. J Forensic Sci. 2011;56: 136–42. doi: 10.1111/j.1556-4029.2010.01539.x 20840295 10.1111/j.1556-4029.2010.01539.x

[34] Reibe S, Madea B. How promptly do blowflies colonise fresh carcasses? A study comparing indoor with outdoor locations. Forensic Sci Int. 2010;195: 52–7. doi: 10.1016/j.forsciint.2009.11.009 20044223 10.1016/j.forsciint.2009.11.009

[35] Sanford MR. Forensic entomology of decomposing humans and their decomposing pets. Forensic Sci Int. Elsevier Ireland Ltd; 2015;247: e11–e17. doi: 10.1016/j.forsciint.2014.11.029 25533575 10.1016/j.forsciint.2014.11.029

[36] Greenberg B. Nocturnal oviposition behavior of blow flies (Diptera: Calliphoridae). J Med Entomol. 1990;27: 807–810. doi: 10.1093/jmedent/27.5.807 2231617 10.1093/jmedent/27.5.807

[37] Goff ML. Comparison of insect species associated with decomposing remains recovered inside dwellings and outdoors on the island of Oahu, Hawaii. J Forensic Sci. 1991;36: 748–753. doi: 10.1520/JFS13085J 1856643

[38] Turchetto M, Vanin S. Forensic entomology and climatic change. Forensic Sci Int. 2004;146 Suppl: S207–9. Available: http://www.ncbi.nlm.nih.gov/pubmed/15639577 doi: 10.1016/j.forsciint.2004.09.064 15639577 10.1016/j.forsciint.2004.09.064

[39] Picard CJ. First record of Chrysomya megacephala Fabricius (Diptera: Calliphoridae) in Indiana, USA. Proc Entomol Soc Washingt. 2013;115: 265–267. doi: 10.4289/0013-8797.115.3.265

[40] Wells JD, Smith JL. First report of *Blaesoxipha plinthopyga* (Diptera: Sarcophagidae) from a human corpse in the USA and a new geographic record based on specimen genotype. J Forensic Sci. 2013;58: 1378–1380. doi: 10.1111/1556-4029.12246 23899435 10.1111/1556-4029.12246

[41] Dahlem GA, Naczi RFC. Flesh flies (Diptera: Sarcophagidae) associated with North American pitcher plants (Sarraceniaceae), with descriptions of three new species. Ann Entomol Soc Am. 2006;99: 218–240. doi: 10.1603/0013-8746(2006)099[0218:FFDSAW]2.0.CO;2

[42] Zehner R, Amendt J, Schütt S, Sauer J, Krettek R, Povolný D. Genetic identification of forensically important flesh flies (Diptera: Sarcophagidae). Int J Legal Med. 2004;118: 245–247. doi: 10.1007/s00414-004-0445-4 15108007 10.1007/s00414-004-0445-4

[43] Stamper T, Dahlem G a., Cookman C, Debry RW. Phylogenetic relationships of flesh flies in the subfamily Sarcophaginae based on three mtDNA fragments (Diptera: Sarcophagidae). Syst Entomol. 2013;38: 35–44. doi: 10.1111/j.1365-3113.2012.00646.x

[44] Pimsler ML, Owings CG, Sanford MR, OConnor BM, Teel PD, Mohr RM, et al Association of *Myianoetus muscarum* (Acari: Histiostomatidae) with *Synthesiomyia nudiseta* (Wulp) (Diptera: Muscidae) on human remains. J Med Entomol. 2016;53: 290–295. doi:http://dx.doi.org/10.1093/jme/tjv203 doi: 10.1093/jme/tjv203 26740477 10.1093/jme/tjv203

[45] Adair TW, Kondratieff BC. Three species of insects collected from an adult human corpse above 3300 m in elevation: a review of a case from Colorado. J Forensic Sci. 2006;51: 1164–1165. doi: 10.1111/j.1556-4029.2006.00236.x 17018102 10.1111/j.1556-4029.2006.00236.x

[46] Norris KR. The Bionomics of Blow Flies. Annu Rev Entomol. 1965;10: 47–68. doi: 10.1146/annurev.en.10.010165.000403

[47] Webmaster H. Climate Pages for College Station (CLL) Houston Intercontinental (IAH) Houston Hobby (HOU) Galveston (GLS). In: National Weather Service Weather Forecast Office—Houston/Galveston, TX [Internet]. 2016 [cited 11 Jul 2016]. Available: http://www.srh.noaa.gov/hgx/?n=climate_graphs

[48] Baumgartner DL. Review of *Chrysomya rufifacies* (Diptera: Calliphoridae). J Med Entomol. 1993;30: 338–352. Available: http://www.ncbi.nlm.nih.gov/pubmed/8459410 8459410 10.1093/jmedent/30.2.338

[49] Disney RHL. Scuttle Flies: The Phoridae. Dordrecht: Springer Netherlands; 1994.

[50] Lesavoy BS, McGaugh SE, Noor MAF. Mitochondrial DNA variation and structure among North American populations of *Megaselia scalaris*. bioRxiv. 2014;pre-print. doi:http://dx.doi.org/10.1101/006288

[51] Denlinger DL, Chen CP, Tanaka S. The impact of diapause on the evolution of other life history traits in flesh flies. Oecologia. 1988;77: 350–356. doi: 10.1007/BF00378041 28311948 10.1007/BF00378041

[52] Bernhardt V, Schomerus C, Verhoff MA, Amendt J. Of pigs and men—comparing the development of *Calliphora vicina* (Diptera: Calliphoridae) on human and porcine tissue. Int J Legal Med. International Journal of Legal Medicine; 2016; 1–7. doi: 10.1007/s00414-016-1487-0 27848012 10.1007/s00414-016-1487-0

[53] Gallagher MB, Sandhu S, Kimsey R. Variation in developmental time for geographically distinct populations of the common green bottle fly, *Lucilia sericata* (Meigen). J Forensic Sci. 2010;55: 438–442. doi: 10.1111/j.1556-4029.2009.01285.x 20102471 10.1111/j.1556-4029.2009.01285.x

[54] Owings CG, Spiegelman C, Tarone AM, Tomberlin JK. Developmental variation among *Cochliomyia macellaria* Fabricius (Diptera: Calliphoridae) populations from three ecoregions of Texas, USA. Int J Legal Med. 2014;128: 709–717. doi: 10.1007/s00414-014-1014-0 24811885 10.1007/s00414-014-1014-0

[55] Catts EP. Problems in Estimating the Postmortem Interval. J Agric Entomol. 1992;9: 245–255. Available: http://scentsoc.org/Volumes/JAE/v9/4/00094245.pdf

[56] Council NR, National Research Council. Strengthening Forensic Science in the United States: A Path Forward [Internet]. Washington, D.C.: National Research Council of the National Academies; 2009. doi:http://www.nap.edu/catalog/12589.html

