# Supplementary material for: Insects and associated arthropods analyzed during medicolegal death investigations in Harris County, Texas, USA: January 2013- April 2016
Source: PLoS One. 2017 Jun 12;12(6):e0179404. doi: 10.1371/journal.pone.0179404 (PMC5467878; doi:10.1371/journal.pone.0179404)
Supplement: S1 Table — Some of the most frequently used taxonomic keys and literature for forensic entomology casework in Harris County, Texas, USA from January 2013 through April 2016. (DOCX) [file pone.0179404.s001.docx]

| **Life Stage** | **Citation** |
| --- | --- |
| Larvae | Seago, J. M. 1953. Fly Larvae: Pictorial key to some common species. US Department of Health, Education and Welfare. Public Health Service, Communicable Disease Center, Training Branch, Atlanta, GA. |
| Larvae | Wells, J. D., J. H. Byrd, and T. I. Tantawi. 1999. Key to third-instar Chrysomyinae (Diptera: Calliphoridae) from carrion in the continental United States. J. Med. Entomol. 36: 638–641. |
| Larvae | Sukontason, K. L., K. Sukontason, and S. Lertthamnongtham. 2002. Surface ultrastructure of third-instar *Megaselia* *scalaris* (Diptera: Phoridae). Mem. Inst. Oswaldo Cruz. 97: 663–665. |
| Larvae | Velásquez, Y., T. Ivorra, A. Grzywacz, A. Martínez-Sánchez, C. Magaña, A. García-Rojo, and S. Rojo. 2013. Larval morphology, development and forensic importance of *Synthesiomyia* *nudiseta* (Diptera: Muscidae) in Europe: a rare species or just overlooked? Bull. Entomol. Res. 103: 98–110. |
| Larvae | Yusseff-Vanegas S. Description of third instars of *Cochliomyia* *minima* (Diptera: Calliphoridae) from West Indies, and updated identification key. J Med Entomol. 2014;51: 1051–1056. doi:10.1603/ME13088. |
| Pupae | Sukontason, K. L., W. Boonsriwong, S. Siriwattanarungsee, S. Piangjai, and K. Sukontason. 2006. Morphology of puparia of *Megaselia* *scalaris* (Diptera: Phoridae), a fly species of medical and forensic importance. Parasitol. Res. 98: 268–272. |
| Adults | Whitworth, T. L. 2006. Keys to the genera and species of blow flies (Diptera: Calliphoridae) of America North of Mexico. Proc. Entomol. Soc. Washingt. 108: 689–725. |
| Adults | Dodge, H. R. 1953. Domestic flies: Pictorial key to common species in Southern U.S., p. 123. In Pict. Keys Arthropods, Reptil. Birds Mamm. Public Heal. Significance. U.S. Department of Health and Human Services, Atlanta, GA. |
| Adults | Pimsler, M. L., T. Pape, S. Johnston, R. A. Wharton, J. J. Parrott, D. Restuccia, M. R. Sanford, J. K. Tomberlin, and A. M. Tarone. 2014. Structural and genetic investigation of the egg and first-instar larva of an egg-laying population of *Blaesoxipha* *plinthopyga* (Diptera: Sarcophagidae), a species of forensic importance. J. Med. Entomol. 51: 1283–1295. |
| Larvae, pupae and adults | Smith, K. G. V. 1986. A Manual of Forensic Entomology, 1st Ed. Cornell University Press, Ithaca, N.Y. |
| Larvae, pupae and adults | Furman, D. P., and E. P. Catts. 1982. Manual of Medical Entomology. Cambridge University Press, New York. |
