# Supplementary material for: Insects and associated arthropods analyzed during medicolegal death investigations in Harris County, Texas, USA: January 2013- April 2016
Source: PLoS One. 2017 Jun 12;12(6):e0179404. doi: 10.1371/journal.pone.0179404 (PMC5467878; doi:10.1371/journal.pone.0179404)
Supplement: S1 File — Results of normality tests of the transformed temperature, relative humidity and estimated post-mortem interval data. Includes Q-Q plots of the data. (PDF) [file pone.0179404.s002.pdf]

Normality Test Transformed Survey Data

Michelle Sanford

2017-04-06

```
> survey_data <-
+   readXL("I:/big survey paper/data for analysis of case survey.xlsx",
+   rownames=FALSE, header=TRUE, na="", sheet="Sheet3", stringsAsFactors=TRUE)
```

```
> survey_data$ln_temperature <- with(survey_data, log(temperature+1))
```

```
> survey_data$ln_RH <- with(survey_data, log(RH+1))
```

```
> survey_data$ln_PMI <- with(survey_data, log(abs_val_PMI+1))
```

```
> with(survey_data, shapiro.test(ln_temperature))
```

```

    Shapiro-Wilk normality test

data:  ln_temperature
W = 0.91515, p-value = 9.28e-09
```

```
> with(survey_data, shapiro.test(ln_RH))
```

```

    Shapiro-Wilk normality test

data:  ln_RH
W = 0.96641, p-value = 0.0004134
```

```
> with(survey_data, shapiro.test(ln_PMI))
```

```

    Shapiro-Wilk normality test

data:  ln_PMI
W = 0.97705, p-value = 0.004886
```

```
> scatterplotMatrix(~ln_PMI+ln_RH+ln_temperature, reg.line=FALSE,
+   smooth=FALSE, spread=TRUE, span=0.5, ellipse=FALSE, levels=c(.5, .9),
+   id.n=0, diagonal = 'qqplot', data=survey_data)
```

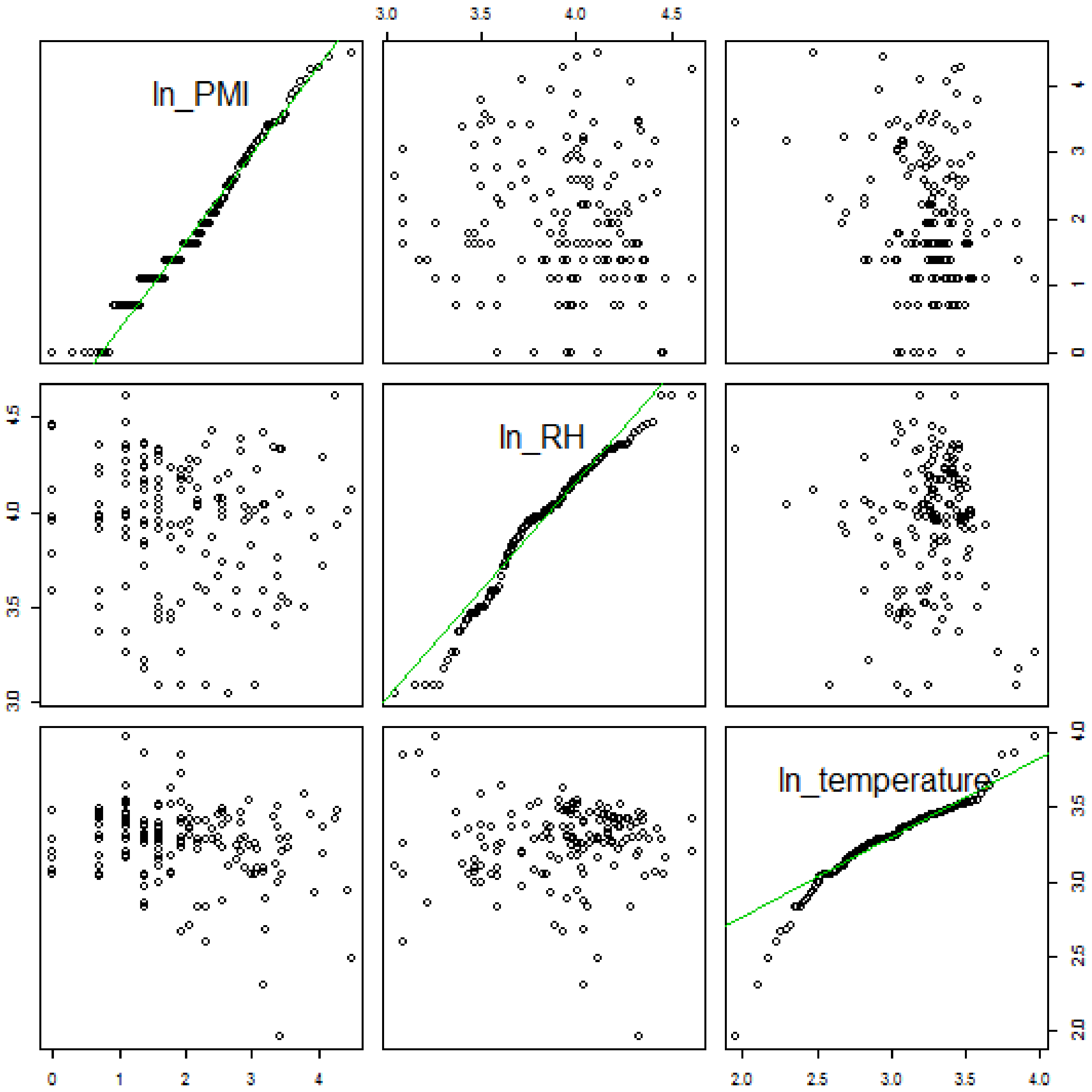

ln_PMI
ln_RH
ln_temperature
