# Supplementary material for: Insects and associated arthropods analyzed during medicolegal death investigations in Harris County, Texas, USA: January 2013- April 2016
Source: PLoS One. 2017 Jun 12;12(6):e0179404. doi: 10.1371/journal.pone.0179404 (PMC5467878; doi:10.1371/journal.pone.0179404)
Supplement: S2 File — The results of the Kruskal-Wallis chi-squared tests on the transformed temperature, relative humidity and estimated post-mortem interval data. (PDF) [file pone.0179404.s003.pdf]

K-W on transformed survey data

Michelle Sanford

2017-04-06

```
> survey_data <- read.table("I:/big survey paper/survey_data.csv",
+   header=TRUE, sep=",", na.strings="NA", dec=".", strip.white=TRUE)
```

```
> with(survey_data, tapply(ln_temperature, location, median, na.rm=TRUE))
```

```
  indoor  outdoor
3.277145 3.397858
```

```
> kruskal.test(ln_temperature ~ location, data=survey_data)
```

```


    Kruskal-Wallis rank sum test

data:  ln_temperature by location
Kruskal-Wallis chi-squared = 6.8397, df = 1, p-value = 0.008916
```

```
> with(survey_data, tapply(ln_temperature, manner, median, na.rm=TRUE))
```

```
    accident     homicide      natural      suicide undetermined
    3.373881     3.421000     3.273357     3.202672     3.363842
```

```
> kruskal.test(ln_temperature ~ manner, data=survey_data)
```

```


    Kruskal-Wallis rank sum test

data:  ln_temperature by manner
Kruskal-Wallis chi-squared = 11.708, df = 4, p-value = 0.01966
```

```
> with(survey_data, tapply(ln_temperature, decomp, median, na.rm=TRUE))
```

```
advanced    early moderate     none skeletal
3.303217 3.290264 3.277145 3.330767 3.437161
```

```
> kruskal.test(ln_temperature ~ decomp, data=survey_data)
```

```


    Kruskal-Wallis rank sum test

data:  ln_temperature by decomp
Kruskal-Wallis chi-squared = 1.9573, df = 4, p-value = 0.7436
```

```
> with(survey_data, tapply(ln_RH, location, median, na.rm=TRUE))
```

```
  indoor  outdoor 
3.970292 4.025192 
```

```
> kruskal.test(ln_RH ~ location, data=survey_data)
```

```


    Kruskal-Wallis rank sum test

data:  ln_RH by location
Kruskal-Wallis chi-squared = 2.7817, df = 1, p-value = 0.09534
```

```
> with(survey_data, tapply(ln_RH, manner, median, na.rm=TRUE))
```

```
    accident     homicide      natural      suicide undetermined 
    3.931826     4.158761     3.951244     3.988813     4.110874 
```

```
> kruskal.test(ln_RH ~ manner, data=survey_data)
```

```


    Kruskal-Wallis rank sum test

data:  ln_RH by manner
Kruskal-Wallis chi-squared = 13.867, df = 4, p-value = 0.007733
```

```
> with(survey_data, tapply(ln_RH, decomp, median, na.rm=TRUE))
```

```
advanced    early moderate     none skeletal 
4.043051 3.988813 3.970292 4.060294 4.007333 
```

```
> kruskal.test(ln_RH ~ decomp, data=survey_data)
```

```
	Kruskal-Wallis rank sum test

data:  ln_RH by decomp
Kruskal-Wallis chi-squared = 3.0391, df = 4, p-value = 0.5513
```

```
> with(survey_data, tapply(ln_TOC, location, median, na.rm=TRUE))
```

```
  indoor  outdoor 
1.945910 1.386294 
```

```
> kruskal.test(ln_TOC ~ location, data=survey_data)
```

```
	Kruskal-Wallis rank sum test

data:  ln_TOC by location
Kruskal-Wallis chi-squared = 3.6595, df = 1, p-value = 0.05575
```

```
> with(survey_data, tapply(ln_TOC, manner, median, na.rm=TRUE))
```

```
    accident     homicide      natural      suicide undetermined 
    1.098612     1.098612     1.945910     2.397895     2.564949 
```

```
> kruskal.test(ln_TOC ~ manner, data=survey_data)
```

```
	Kruskal-Wallis rank sum test

data:  ln_TOC by manner
Kruskal-Wallis chi-squared = 25.338, df = 4, p-value = 4.302e-05
```

```
> with(survey_data, tapply(ln_TOC, decomp, median, na.rm=TRUE))
```

```
 advanced     early  moderate      none  skeletal 
2.8332133 1.0986123 1.6094379 0.6931472 3.8162006 
```

```
> kruskal.test(ln_TOC ~ decomp, data=survey_data)
```

```
    Kruskal-Wallis rank sum test

data:  ln_TOC by decomp
Kruskal-Wallis chi-squared = 71.849, df = 4, p-value = 9.238e-15
```
