# Supplementary figures and images for: Insects and associated arthropods analyzed during medicolegal death investigations in Harris County, Texas, USA: January 2013- April 2016
Source: PLoS One. 2017 Jun 12;12(6):e0179404. doi: 10.1371/journal.pone.0179404 (PMC5467878; doi:10.1371/journal.pone.0179404)

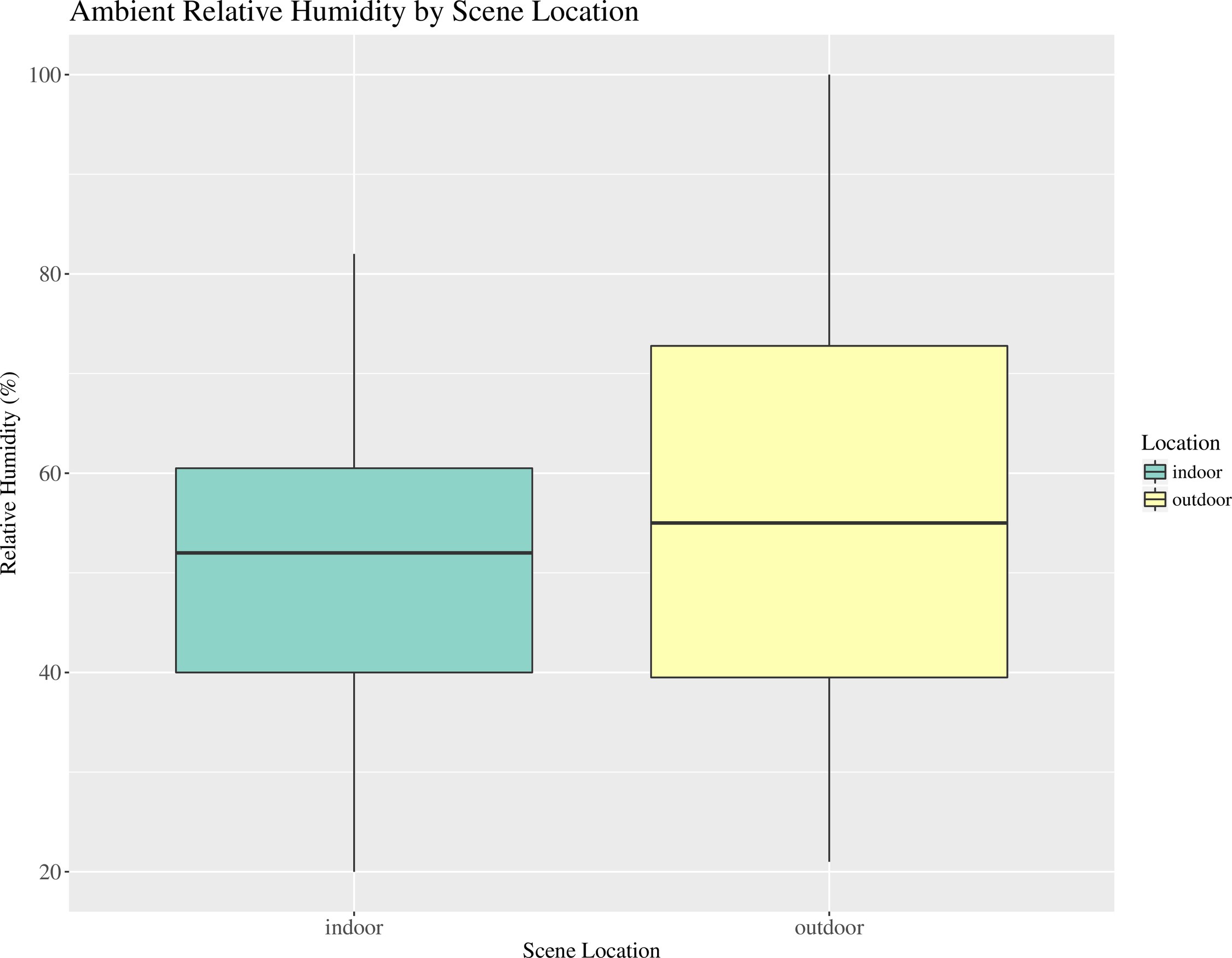

Supplement: S1 Fig — Boxplot illustrating the median and range of observed relative humidity for indoor and outdoor scene locations. (TIF) [file pone.0179404.s005.tif]

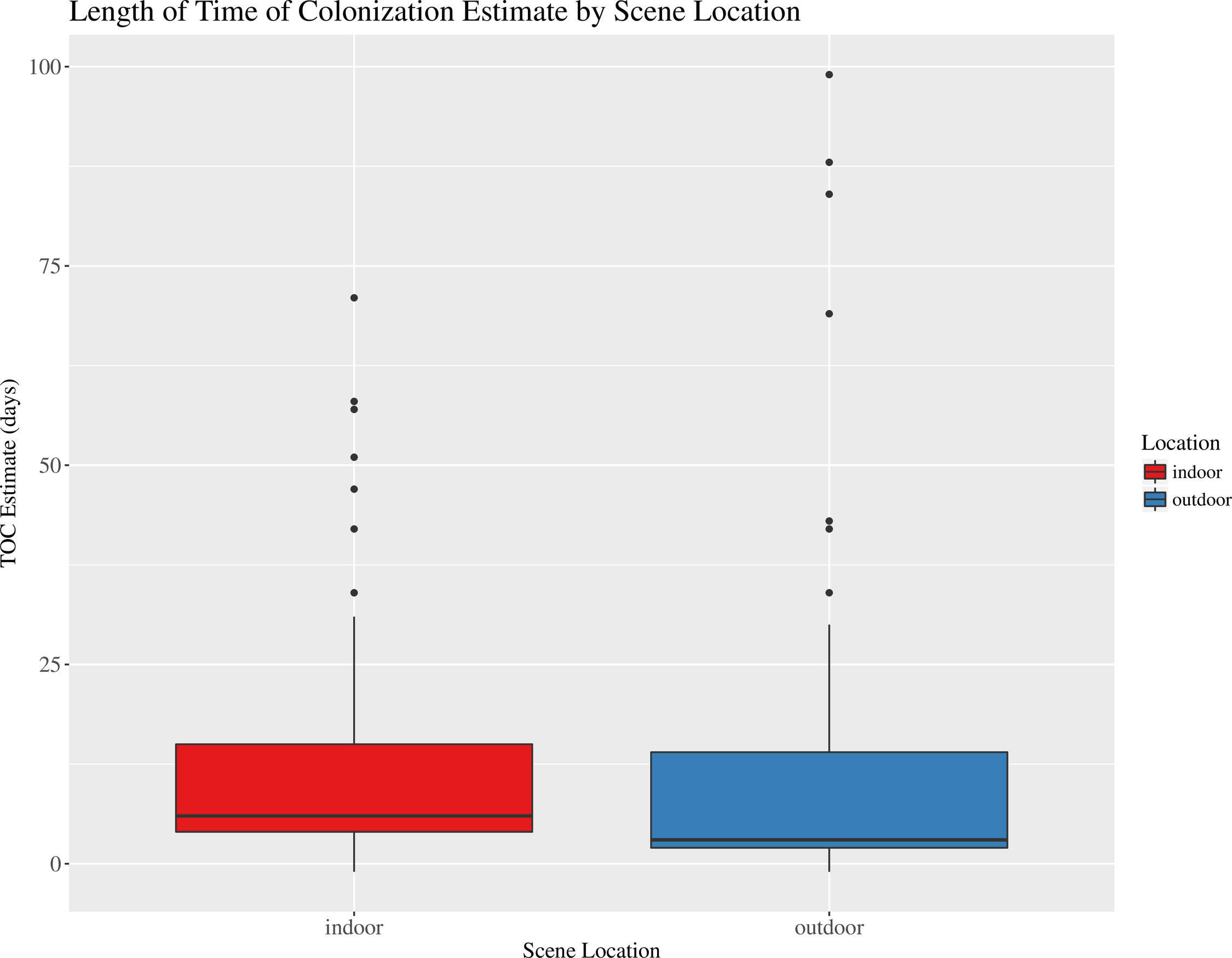

Supplement: S2 Fig — Boxplot illustrating the median and range of the length of time of colonization estimates (days) by scene location (indoors vs. outdoors). (TIF) [file pone.0179404.s006.tif]

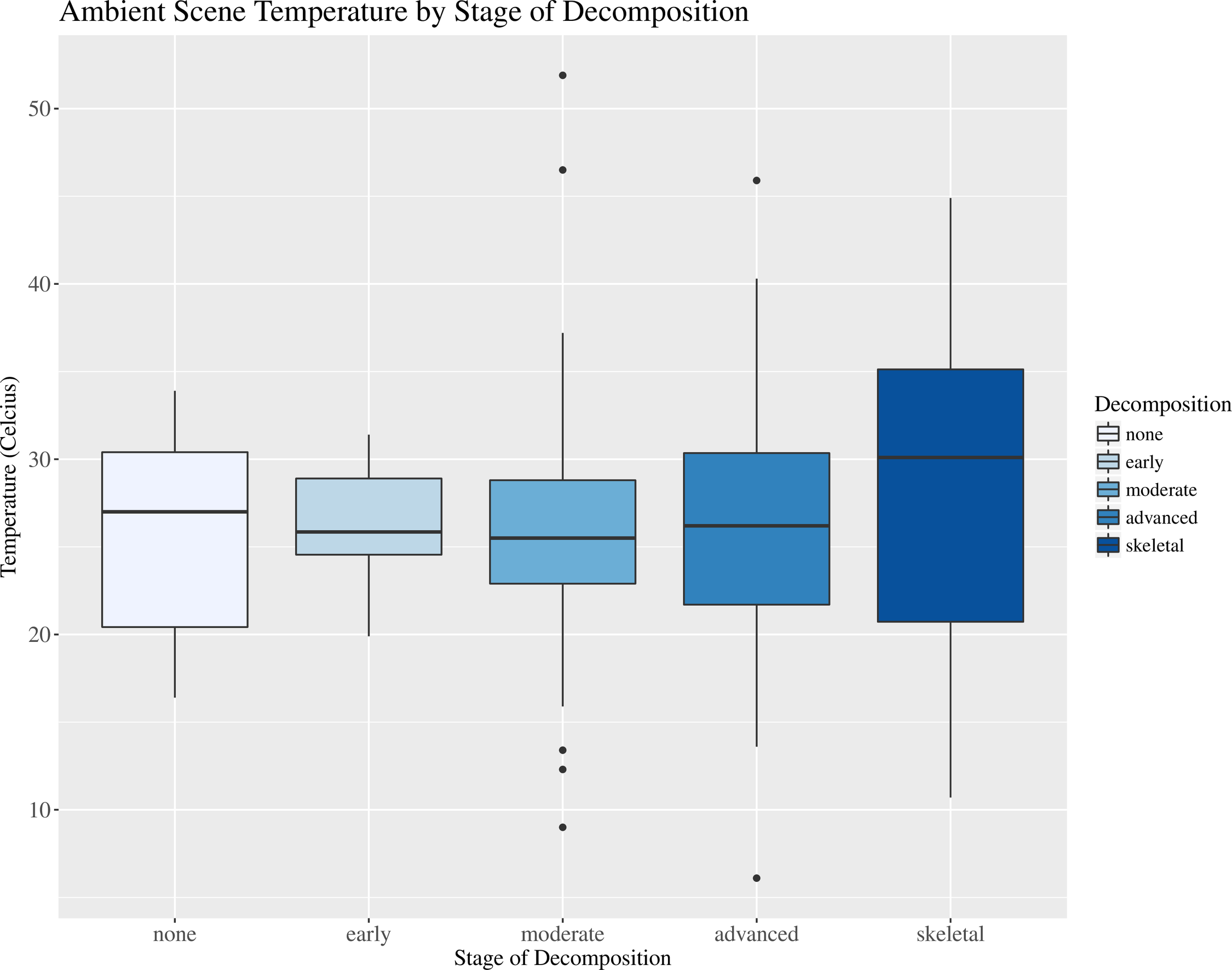

Supplement: S3 Fig — Boxplot illustrating the median and range of the observed ambient scene temperatures (°C) by the observed stage of decomposition for the decedent. (TIF) [file pone.0179404.s007.tif]

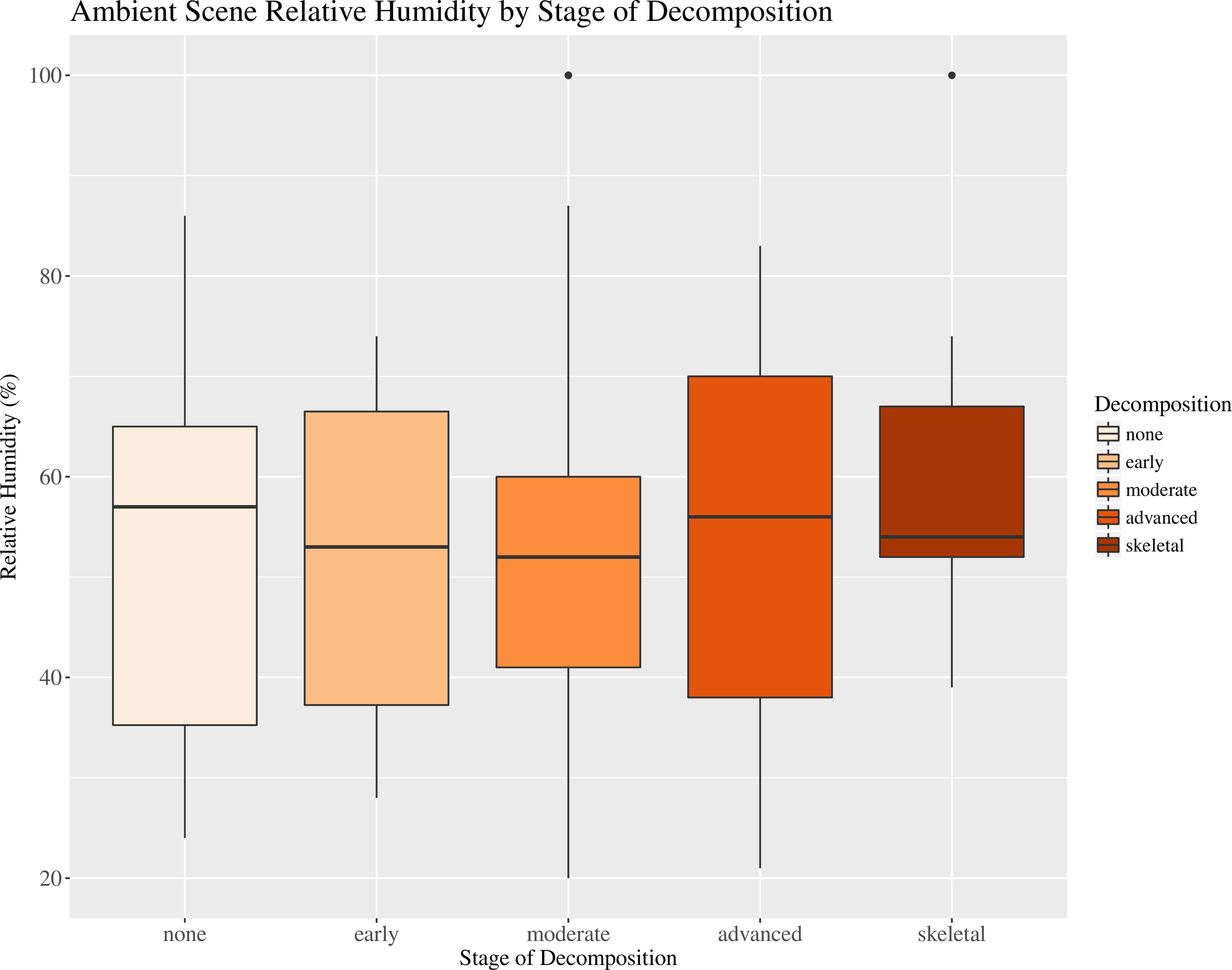

Supplement: S4 Fig — Boxplot illustrating the median and range of observed relative humidity at scene by the observed stage of decomposition for the decedent. (TIF) [file pone.0179404.s008.tif]
